# An Adapted Similarity Kernel and Generalized Convex Hull for Molecular Crystal Structure Prediction

**DOI:** 10.1021/acs.cgd.5c01220

**Published:** 2025-10-23

**Authors:** Jennie Martin, Michele Ceriotti, Graeme M. Day

**Affiliations:** † School of Chemistry and Chemical Engineering, 7423 University of Southampton, Southampton SO17 1BJ, U.K.; ‡ 27218 École Polytechnique Fédérale de Lausanne, CH-1015 Lausanne, Switzerland

## Abstract

We adapted an existing approach to identifying stabilizable crystal structures from prediction sets—the Generalized Convex Hull (GCH)—to improve its application to molecular crystal structures. This was achieved by modifying the Smooth Overlap of Atomic Positions (SOAP) kernel to define the similarity of molecular crystal structures in a more physically motivated way. The use of the adapted similarity kernel was assessed for several organic molecular crystal landscapes, demonstrating improved interpretability of the resulting machine-learned descriptors. We also demonstrate that the adapted kernel results in improved performance in predicting lattice energies using Gaussian process regression. Our overall findings highlight a sensitivity of similarity kernel-based landscape analysis methods to kernel construction, which should be considered when applying these methods.

## Graphical abstract

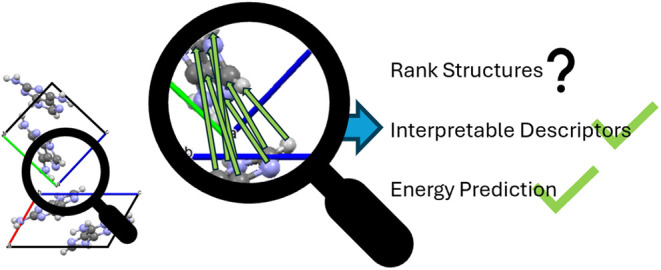

## Introduction

The prediction of plausible crystal structures of a molecule, prior to crystallization or even prior to molecular synthesis, can expedite the materials discovery process. Functional properties of a material, including stability, porosity,^1,2^ conductivity,^3,4^ solubility,^5^ and compactability^6^ can all be impacted by the crystal packing. Therefore, molecular crystal structure prediction (CSP) facilitates property prediction and can be used to prioritize the most promising candidate molecules for experimental screening. Furthermore, CSP searches for potential crystal structures can add assurance to polymorph screening,^7–9^ reducing the risk of late appearing polymorphs, which can have important consequences for the control of properties of pharmaceutical materials.^5^

At their core, most approaches to molecular CSP involve two steps, with generation of trial crystal structures to sample structure space followed by geometry optimization of those structures to identify local minima on a structure-energy landscape.^10,11^ Each local minimum is assumed to correspond to a possible observable crystal structure. These methods have been successfully applied for polymorph screening^7–9^ and in guiding the discovery of functional organic^1,2,12^ and inorganic^13,14^ materials. However, common approaches to molecular CSP still suffer key limitations, including the “overprediction problem”: for any given molecule, most CSP methods predict many more crystal structures (local energy minima) than the number of true polymorphs of a system that are likely to be found experimentally.^15^ There are likely several contributing factors leading to the discrepancy, including neglect of thermal effects in smoothing the energy surface^16^ and the role of kinetics in determining which structures are observed.^15^ Another key factor is that many of the local minima will not be thermodynamically competitive with the true global energy minimum crystal structure under the conditions used for crystallization.^15^ Therefore, the enumeration of local minima is inherently overpredictive and so some filtering is needed to identify the most likely stabilizable structures from the prediction sets.

It is commonly assumed that the structures in a low-energy region of the predicted landscape, defined by some cutoff above the global energy minimum, are those likely to be sufficiently stable to be observable. One justification for an energy cutoff in predicting crystal structures is the range of calculated energy differences between known polymorphs: a large-scale computational study showed that 95% of observed polymorphs are separated by less than 7.2 kJ/mol.^16^ A recent CSP study of over 1000 small molecules provides further empirical validation of applying an energy cutoff to structure prediction results: 74% of known crystal structures were located within 2 kJ mol^–1^ of the global energy minimum and 98% within 8 kJ mol^–1^.^17^ Therefore, it is highly likely that the known or discoverable crystal structures of a molecule will lie within a small lattice energy window above the global minimum on the predicted landscape. However, limiting consideration to crystal structures with low relative lattice energy suffers a lack of generalizability, as additional stabilization factors can lead to the realization of crystal structures with higher lattice energy. For example, several studies have found porous, low-density, high-energy structures, lying in “spikes” on an energy-density CSP landscape.^1,2,18–20^ These structures, sometimes occurring further than 50 kJ mol^–1^ above the global minimum,^19^ can be stabilized during crystallization by the inclusion of solvent within their pores. Therefore, approaches to identifying the most promising subsets of predictions based solely on lattice energy are not always appropriate. For systems anticipated to be porous, one approach is to select as likely candidates the crystal structures lying near the “leading edge” of the energy-density landscape^1,2^ (Figure 1a).

**Figure 1:**
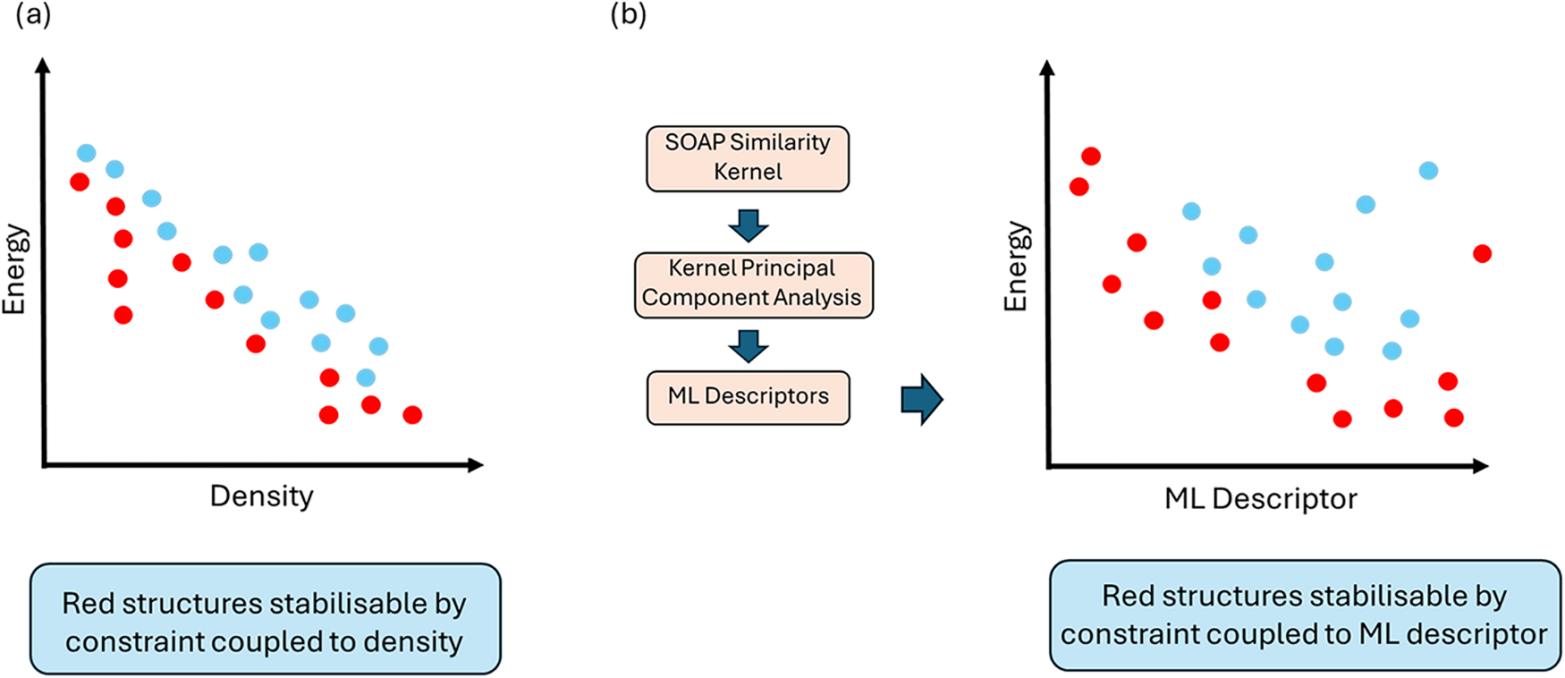
Conceptual CSP landscape showing structures (highlighted in red) that would be identified as stabilizable by different approaches. In (a), predicted structures are plotted according to their energy and density, and the red structures are those close to the leading edge along that landscape. These may be stabilizable by some constraint coupled to density. In (b), structures are plotted according to their energy and values of a machine-learned descriptor—derived from a SOAP kernel. Structures in red are those on or close to a convex hull on that landscape. They may be stabilizable by some constraint coupled to the ML descriptor.

High energy predicted crystal structures can have attractive properties. Therefore, a more generalizable approach to identifying experimentally realizable structures would be valuable, considering the possibility of stabilizing structures not just by constraints related to density, such as solvent inclusion, but also stabilization by other constraints—which could relate to other structural features. This would be akin to identifying as stabilizable structures lying along the leading edge of a landscape of energy and some other structural descriptor.

The Generalized Convex Hull (GCH)^21^ is an approach to identify stabilizable crystal structures from prediction sets; convex hull methods, used frequently in inorganic materials discovery,^22,23^ identify predicted crystal structures lying close to a “convex hull” across the low-energy edge of a landscape as stabilizable. However, while convex hulls are traditionally constructed from the distribution of energy vs manually selected features such as composition or molar volume, the GCH automates the identification of suitable structural features against which the convex hull is determined. The machine-learned (ML) descriptors are derived from a kernel principal component analysis (kPCA) of a Smooth Overlap of Atomic Positions (SOAP) similarity kernel, so as to be optimal in that they capture maximal structural variance across the prediction set. Their derivation via an additive atomic environment descriptor, SOAP, allows the corresponding kernel to pick out key structural information and so the derived descriptors may relate to more concrete physical features, such as density or molecular conformation. The predicted structures on or close to the hull are then said by the approach to be stabilizable with respect to some experimental constraint that couples to the descriptors used to construct the hull^21^ (Figure 1b). While the GCH removes the researcher bias of manually selecting features for hull construction, there remains a dependency of results upon the number of ML descriptors used in its construction, as well as the parameters of the initial descriptors and/or kernel construction.^21^

SOAP^24^ describes the environment about each atom from the distribution of surrounding Gaussian smoothed atomic densities out to a cutoff radius. The local kernel describing the similarity between two atomic environments is given by the dot product of the vectors of the concatenated partial power spectra which describe the atomic density of each pair of atomic species in the environment—expanded in a basis of spherical harmonics and radial basis functions

1 $$k\left(A_{i},B_{j}\right)=\rho \left(A_{i}\right)\cdot \rho \left(B_{j}\right)$$

where **ρ**(*x*) has components

2 $$\rho {\left(x\right)}_{b_{1}b_{2}l}^{\alpha \beta }=\pi \sqrt{\frac{8}{2l+1}}\sum_{m}\left({\left(c_{b_{1}\mathrm{lm}}^{\alpha }\right)}^{†}c_{b_{2}\mathrm{lm}}^{\beta }\right)$$

with *b*_1_, *b*_2_, *m*, and *l* indexing basis functions of the expansion and the corresponding coefficients *c*_*blm*_^*s*^ being taken from the description of the atomic density of species *s* in the environment.^25^

Global kernels defining the similarity of two sets of atoms can then be defined via combination of the local kernels between atoms in the respective structures. Several conventional global kernels have been applied to describe the similarity of crystal structures, all of which consist of some function—however complex—of all atom–atom similarities between atoms in the unit cells of the respective crystal structures. The simplest global kernel, the average kernel, evaluates the similarity between two crystal structures as the arithmetic mean of all atom–atom similarities for all pairs of atoms between the unit cells of the two structures. A more complex kernel that has been used is the ReMatch kernel, in which the global similarity between two crystal structures combines the average of atom–atom similarities between atom pairs corresponding to a “best-match assignment” of the atoms between unit cells with a weighted contribution from the simple average SOAP kernel. For a given structure set, the resulting kernel matrix—which gives the similarity of each structure pair—is usually then normalized such that the self-similarities are equal to one.^25^

These constructions of the global kernel, which have been applied in prior applications of the GCH,^21^ are not well suited to molecular crystal structures because they consider atom pairs that occupy distinct intramolecular environments that cannot be interchanged between structures without breaking covalent bonds. The corresponding local kernels should not meaningfully contribute to the quantification of similarity of molecular crystal structures, where the aim is to study the crystal structure landscape of a fixed molecule. However, conventional global kernels fall short of explicitly excluding these local kernels; their inclusion in the simple average kernel is clear—all pairwise local kernels are included, without restriction. More mindful constructions, such as the best-match or ReMatch constructions, determine the contribution of each local kernel by maximizing the average similarity^25^ without using chemical considerations to limit the possible atom–atom pairwise comparisons. Thus, it is not ensured that the optimized pairing scheme matches only atoms that correspond to the same atom of the underlying molecule. That is, it is not guaranteed that the best-match/ReMatch kernel constructions exclude unreasonable local kernels.

With these considerations in mind, we sought to develop a global SOAP kernel for molecular crystal structures, which directly excludes comparisons between chemically inequivalent atoms *a priori*. The construction only includes comparisons between atoms that can, when considering molecular symmetry, be said to correspond to the same atom of the underlying molecule; we refer to such atom pairs as “analogous atoms”. We then tested the utility of this kernel construction via its implementation in the GCH, including investigation of the interpretability of the machine-learned features, and by assessment of the kernel’s performance in lattice energy predictions using Gaussian Process Regression.

## Developing the Kernel

### Constraining Comparisons

Quantifying the similarity of a pair of molecular crystal structures requires a comparison of the environments of each molecule in the asymmetric unit of each crystal structure. Atom-atom comparisons only meaningfully contribute to the description of this similarity if they correspond to the same, or symmetry equivalent, atom of the underlying molecule. Recall that the average global SOAP kernel, which simply takes the overall similarity between two crystal structures to be the arithmetic mean of all atom–atom similarities for all pairs of atoms between the unit cells of the two structures, does not impose this meaningful restriction. Therefore, a new, adapted, kernel construction is required. Therefore, in constructing a new global SOAP kernel by adapting the construction of the average global SOAP kernel, the similarity between two crystal structures is defined as the similarity between analogous atoms of the two crystal structures—averaged across all atoms (and their corresponding analogous partners) in the molecule. This construction therefore is similar to the average global SOAP kernel—albeit with physically motivated constraints upon the atom–atom similarities included in the average

In the simplest case, for molecules of point group *C*_1_, analogous atoms can be identified as those that share the same molecular atom index after ensuring that atom indexing is consistent between crystal structures. For structures with one molecule in their asymmetric unit (*Z*′ = 1), the kernel *K*(*A*,*B*) between crystal structures *A* and *B* is given by

3 $$K\left(A,B\right)=\frac{\sum_{i=0}^{N-1}\sum_{j=0}^{N-1}k\left(A_{i},B_{j}\right)\times \delta_{\mathrm{ij}}}{\mathrm{stN}}$$

where *i* and *j* index the atom within the molecule, δ_*ij*_ is the Kronecker delta, *N* is the number of atoms within the molecule, and *s* and *t* denote the number of copies of the asymmetric unit within crystal structures *A* and *B* respectively. Practically, the comparisons need only be run for one copy of the asymmetric unit—as the corresponding comparisons for other copies of the asymmetric unit within the crystal structures will return the same result. Then, *s* and *t* can be set equal to one.

Comparisons between crystal structures with multiple copies of the molecule in the asymmetric unit (*Z*′ > 1) should consider the similarities between the environments of all molecules of the respective asymmetric units and the kernel should deterministically combine the similarity information. In this work, we used an averaging scheme: a given analogous atom–atom comparison, *K*(*A*_*i*_,*B*_*j*_), is made between all pairs of molecules of the respective asymmetric units and the overall similarity for the comparison is taken to be the average of these contributions. The analogous atom–atom comparisons are then combined as before, leading to

4 $$K\left(A,B\right)=\frac{\sum_{i=0}^{N-1}\sum_{j=0}^{N-1}k\left(A_{i},B_{j}\right)\times \delta_{\mathrm{ij}}}{\mathrm{sStTN}}$$

where *S* and *T* denote the *Z*′ of crystals *A* and *B*.

### Molecular Symmetry

When a molecule has symmetry beyond the identity operation we must also consider pairs of atoms that are symmetry equivalents under some molecular point group operator, so have identical intramolecular environments in a given conformation of the molecule. This is necessary to avoid being restricted by the given molecular atom indexing which can only be considered consistent and meaningful up to a transformation by a point group operator. Where a molecular point group operator maps one atom in the molecule to another, we refer to this as an atom–atom mapping.

We consider the action of each molecular point group operator in turn, constructing a kernel *K*(*A*,*B*)_*q*_ for each operator *q* within the point group of the molecule. Each such kernel includes only comparisons between atoms that are considered analogous under the action of *q*. For *Z*′ = 1

5 $$K{\left(A,B\right)}_{q}=\frac{\sum_{i=0}^{N-1}\sum_{j=0}^{N-1}k\left(A_{i},B_{j}\right)\times g\left(i,j\right)}{\mathrm{stN}},g\left(i,j\right)=\{\begin{array}{l} 1,\mathrm{if}\;q\left(i\right)=j\\ 0,\mathrm{otherwise} \end{array}$$

To account for all reasonable comparisons, such kernels are constructed for all relevant atom–atom mappings and combinations of mappings. By the closed property of groups, those combinations of mappings for a given molecule in a given point group also correspond to an operator within the point group. The examples in this paper used only structure sets in which the molecule was either asymmetrical, or was treated as rigid—and so only one conformation of the molecule was present in the structure set. Therefore, only one point group and corresponding set of atom–atom mappings *q* had to be considered to identify all valid mappings for the structure set. We have developed a more complex approach, incorporating the direct product of groups, to identify the sets of mappings for structure sets of flexible, symmetrical molecules, but this is not discussed here.

The kernels *K*(*A*,*B*)_*q*_ calculated for each mapping are averaged such that the kernel between any two crystal structures is taken to be the mean of the individual similarity values for that structure pair across those kernels

6 $$K\left(A,B\right)=\frac{\sum_{q=1}^{Q}K{\left(A,B\right)}_{q}}{Q}$$

where *Q* is the total number of point group operators.

The average is used here rather than the maximum to guarantee a positive semidefinite kernel, which is required in some applications, such as Gaussian Process Regression^26^ for energy prediction.^27–29^ This property is not maintained, however, when working with structure sets featuring both varying *Z*′ and symmetric underlying molecules. Consequently, the current construction is applicable only to structure sets of consistent *Z*′ or to any structure set where the underlying molecule is asymmetrical. It may be that comparisons that include different numbers of atoms between structures being compared cause issues in this context. It may be possible to address this through replication of atomic environments so as to reach a common multiple of atom counts, however, to do so would significantly increase the computational cost and has not been tested here.^25^

## Measures of Kernel Performance

### Identifying Stabilizable Structures

Structures are identified as potentially stabilizable using the GCH approach based on their proximity to the hull: structures closer to the hull are more likely to be stabilizable. Therefore, when using the GCH to narrow down a set of predicted structures for further investigation, we select a subset of the structures, referred to as the “candidate pool”, within an energy range of the hull.

For organic molecular crystals, there is not an established measure of the energy window relative to the hull in which it is good practice to search for stabilizable structures. We trust in the knowledge of the existing polymorphs to define the candidate pool for each system. The three molecules chosen to study candidate pools (Table 1) have been thoroughly studied, so we define the candidate pool here to be the number of structures in the smallest dressed energy window (the energy window relative to the hull) that contains all currently known polymorphs—or rather the structures in the set that correspond to those polymorphs. The predicted structures corresponding to known polymorphs are identified as discussed in SI Section 1.

**Table 1:** Systems Explored in This Work, Their Function, and the Kernel Assessment Metrics for Which the Predicted Structure Sets Have Been Used^a^

System	Function	Metrics	Known Polymorphs
*2,6-diaminopurine (DAP)	Porous Material/Pharmaceutical^30^	Descriptor interpretability	3 fully characterized (1 in data set)
ROY^31^	Pharmaceutical Precursor^32^	Descriptor interpretability & Candidate Pools	14
*Galunisertib^7^	Potential pharmaceutical^7^	Descriptor interpretability & Candidate Pools	10 (9 in data set)
triptycenetrisbenzimidazolone (TTBI)^2^	Porous Material^1^	Descriptor interpretability & Candidate Pools	5
Trimesic Acid^18^	Functional material/Porous Material^18^	Descriptor interpretability	3
*Chlorpropamide^17^	Pharmaceutical^17^	Energy prediction	9 (8 in data set)

a ROY is the name commonly given to 5-methyl-2-((2-nitrophenyl)­amino)­thiophene-3-carbonitrile, based on its red, orange and yellow polymorphs. *These available structure sets did not contain all known polymorphs. Explanations of this incompleteness can be found in SI Section 3.

Comparing the candidate pools arising from the GCH when implementing different kernels provides a useful means for assessing the effectiveness of the respective kernels for stabilizable structure identification. For the purposes of a materials discovery workflow, assuming that a given candidate pool can be taken to include all stabilizable structures (i.e., assuming no false negatives) then it is desirable to have as small a candidate pool as possible (minimal false positives) in order to conserve effort in further experimental or computational work on the predictions. Therefore, as a performance metric, we seek minimal candidate pools.

### Interpreting Descriptors

The ML descriptors derived via kPCA using a SOAP kernel are somewhat abstract and the kPCA component weightings are not directly interpretable. However, an understanding of the meaning of the ML descriptors can be gained by identifying intuitive structural features to which they relate, giving a picture of the structural features being picked out by the SOAP kernel and kPCA decomposition.

Intuitive interpretations of the ML descriptors are useful for a materials discovery workflow. The experimental constraints that must be controlled in order to selectively stabilize the near-hull structures couple to the descriptors used to construct the hull.^21^ As such, if an intuitive feature(s) can be found that closely aligns with the ML descriptor(s) used in hull construction, then a knowledgeable researcher could propose coupled constraints, thus guiding the crystallization variables considered in crystal form screening. A familiar example of coupled descriptors and experimental constraints is seen in the stabilization of crystal structures of different densities by controlling the pressure. High pressure has, for example, been used to crystallize polymorphs that are not stable at low pressure.^8^ Using the GCH approach—alongside interpretation of the ML descriptors—would allow for retroactive identification of the intuitive descriptors that are “optimal” alongside the corresponding set of stabilizable structures.

Crucially though, interpretability of the ML descriptors also gives assurance that the underlying atomic environment descriptors and kernel construction are reasonable and able to identify physically/chemically meaningful features. Therefore, we explore the interpretability—the clarity and strength of their relationship to intuitive features—of the highly ranked ML descriptors when comparing kernels.

### Energy Prediction

A useful application of a similarity kernel is in lattice energy prediction for reranking sets of predicted structures, by training ML models to predict energies from high levels of theory, but with greatly reduced computational effort. Gaussian Process Regression (GPR) has been employed in this way,^27^ for example achieving lattice energy prediction errors <1 kJ/mol for crystal structures of pentacene.^29^ More recently, a multifidelity GPR learning approach has been demonstrated for CSP, exploiting correlations between lattice energies predicted at different levels of theory to further reduce the number of highest level energy calculations required, while increasing accuracy of the predicted energies.^28^

Lattice energy prediction by GPR relies on the kernel capturing physically meaningful structural similarities between crystal structures. Therefore, performance in GPR energy prediction is a useful measure of the utility and reasonable construction of the adapted and average SOAP kernels.

## Results and Discussion

### Systems of Interest

Different systems act as good test cases for assessing different aspects of kernel performance. For instance, it is more powerful to compare candidate pools for systems with multiple known polymorphs. Table 1 notes the systems that have been investigated and the kernel success metrics which have been explored using the data for those systems. All CSP structure sets used have been obtained from the cited literature, with the exception of DAP, whose CSP was performed specifically for this work (see SI Section 2 for DAP CSP methodology).

### Identifying Stabilizable Structures

Figure 2 compares the size of the candidate pools as derived from the GCH using different kernels. Results for galunisertib do not consider Form I—for which a match could not be found in the predicted structure set. Recall that, here, a smaller candidate pool is a preferable result, as this reduces the time and resources needed for further calculation or experimental testing on potential candidates.

**Figure 2:**
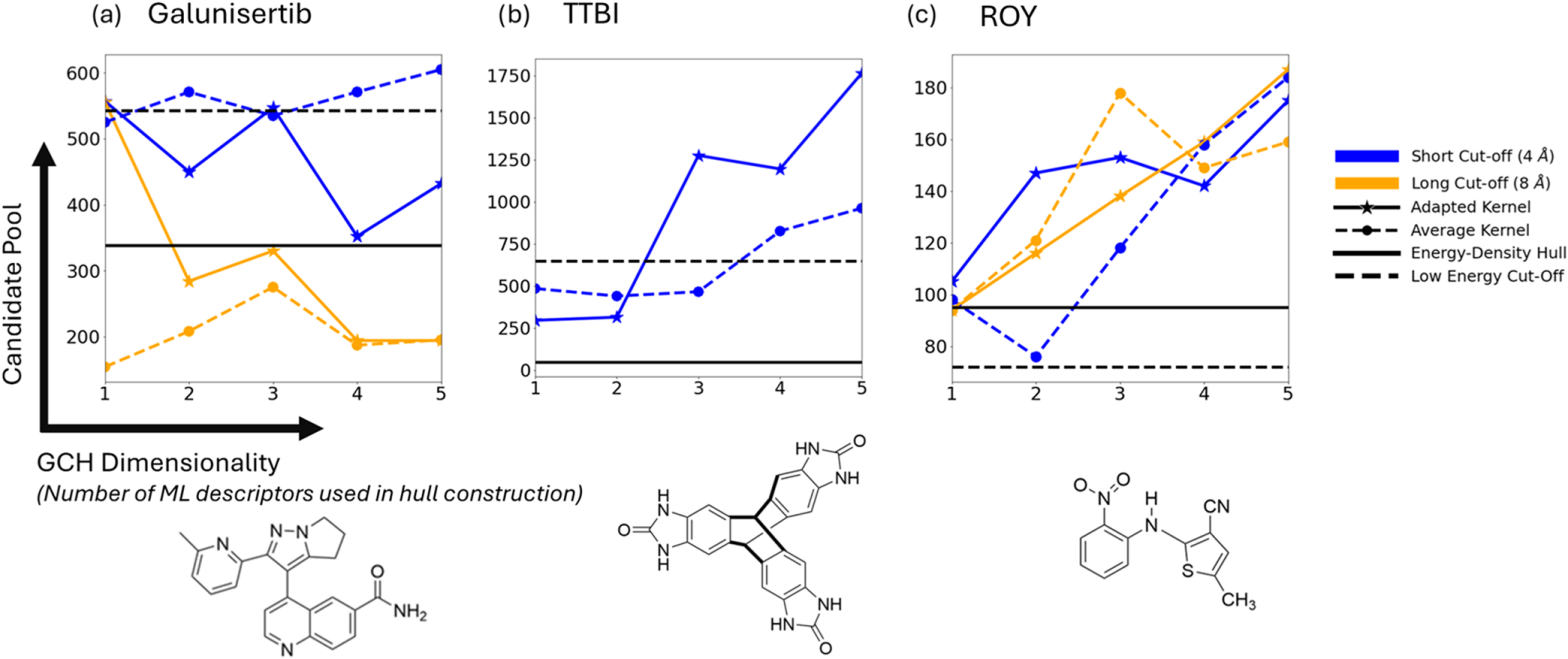
Candidate pools for each system as a function of hull dimensionality, SOAP cutoff, and kernel construction for each. Different curves denote different kernel constructions—with different kernel types and underlying SOAP cutoffs. Horizontal bars indicate the candidate pools for each system as calculated via traditional landscape analysis methods. Data is shown for the prediction sets of (a) galunisertib, (b) TTBI, and (c) ROY.

The results indicate a dependence of the candidate pools upon the dimensionality of the GCH (the number of kPCA eigenvectors used to construct the hull), and SOAP cutoff radius. This complicates the comparison between kernels, as the relative performance of the two kernels is also dependent upon these factors. For example, in the case of TTBI (Figure 2b) the adapted kernel selects smaller candidate pools when few ML descriptors are used to construct the hull, but at higher dimensionalities its performance significantly deteriorates and the average kernel appears superior. Additionally in the case of galunisertib (Figure 2a), the adapted kernel outperforms the average at short cutoff radii (4 Å) but at long cutoff (8 Å) the converse is seen.

It is also important to compare the effectiveness of the GCH to more traditional approaches of selecting important structures from CSP landscapes: candidate pools based on a relative energy cutoff (dashed black lines, Figure 2) or an energy-density convex hull (solid black lines). Galunisertib and TTBI both have observed polymorphs that are accessed experimentally by desolvation of crystal structures that incorporate solvent molecules when crystallized.^1,7^ Desolvation can leave the crystal structure trapped in a metastable local energy minimum, so that the polymorphs accessed by desolvation often correspond to high-energy structures on CSP landscapes. For both molecules, GCH candidate pools are generally smaller than candidate pools from an energy cutoff, with the exception of some candidate pools selected from high dimensional GCH constructions. These results highlight the importance of considering structural features, as well as calculated lattice energies, in identifying candidate polymorphs from CSP studies; the candidate pool results demonstrate that the GCH identifies relevant structural features in a data-driven manner. For TTBI, using an energy-density convex hull for selecting the candidate pool is more effective than the GCH constructed from either kernel, reducing 647 candidate pool structures using an energy cutoff to 48 structures (Figure 2b), likely due to the known strong dependence of stability upon density and its coupled constraints, such as solvent inclusion, for that system. For galunisertib, the candidate pool from an energy-density convex hull is smaller than from a GCH using either kernel with a short SOAP cutoff distance. However, when the GCH is based on SOAP with a longer distance cutoff, the candidate pools are mostly smaller than from the energy-density convex hull. For ROY (Figure 2c), an energy cutoff produces the smallest candidate pools and the GCH generally produces larger candidate pools than an energy cutoff or energy-density candidate pool.

It is useful to note, however, that both traditional and GCH approaches to identifying stabilizable structures have been impactful for the tested cases. Full CSP landscapes, prior to filtering for low-energy structures, can contain thousands or even tens of thousands of predicted local minima and so even the larger candidate pools seen in this work could correspond to significant reductions in the size of the structure sets. Energy-density plots for the full available CSP landscapes of the galunisertib, TTBI, and ROY systems can be found in SI Section 4.

The candidate pools shown here for any given method are those necessary to capture all known structures in the prediction set. For some applications, where succeeding computational or experimental processes are costly, it may be that the number of allowed candidates is particularly restricted—making testing on some of these candidate pools unfeasible. It is therefore of interest to see the recovery of known structures possible by different methods as the pool size taken increases. Figure 3 shows the percentage of known structures lying in pools of different sizes for each system—drawing from the energy cutoff and energy-density convex hull methods, as well as for a single GCH construction (1D, 4 Å cutoff) for each kernel type. These tests indicate that the overall candidate pool figures may not represent a complete picture of the relative promise of different methods—particularly when resources are limited. For example, in Figure 3c, as the candidate pool taken increases, the GCH approach (average or adapted kernel) capturing the highest percentage of the known structures changes. We additionally explored the potential of trends in the “order” in which known structures are captured by the candidate pools of increasing sizes. By comparing the rankings (SI Section 5) of the known polymorphs as determined by the GCH constructions to those rankings based purely on energy, we found some indication that lower energy known structures will be captured by smaller GCH candidate pools, and higher energy known structures may require larger GCH candidate pools. We calculated the kendall rank correlation between energetic rankings and GCH-based rankings of known structures for the galunisertib, TTBI, and ROY systems using 1D GCH constructions with a 4 Å cutoff. For the cases of galunisertib and ROY, there were significant correlations—with coefficients in the range of 0.48 to 0.87. However, for the case of TTBI there was no significant correlation (see SI Section 5 for full details). Therefore, we conclude that it may be possible to capture the most thermodynamically stable polymorphs within smaller GCH candidate pools, but this cannot be universally assumed. It is also pertinent here to note that this finding is not unexpected. As energy is itself used as a variable in the hull construction, low-energy points will commonly lie close to the hull and so be recoverable within small candidate pools. Additionally the global minimum prediction in any data set will then by construction lie on the hull calculated for that data set—and so any known polymorph corresponding to the global minimum prediction will be classed as the most stable by both purely energetic and GCH-based rankings.

**Figure 3:**
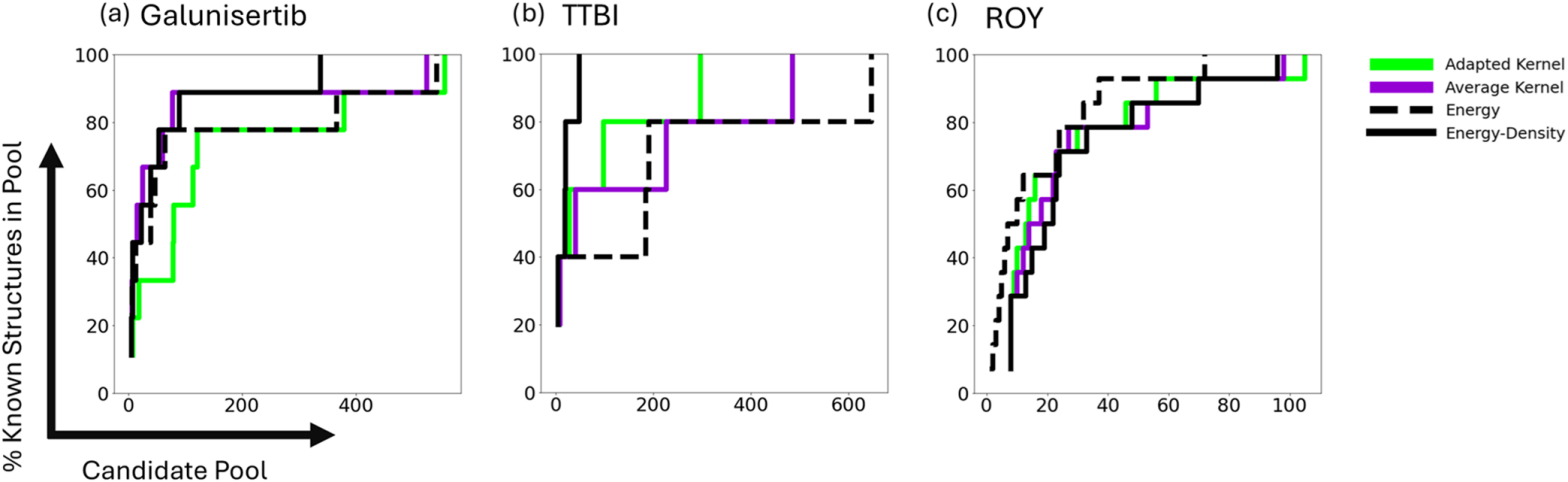
Step plots indicating the percentage of known structures lying in candidate pools of different sizes for different methods of pool selection. Methods, denoted by color and line style, are the energy cutoff and energy-density convex hull methods, as well as GCH constructions (1D, 4 Å cutoff) for each kernel type. Data is shown for the prediction sets of (a) galunisertib, (b) TTBI, and (c) ROY.

Given the inconsistent relative performance of the kernels, we also explored whether the observed differences in total candidate pools are significant. This is because one explanation for the inconclusive results seen could be that the performance differences are merely due to chance—and fall within the region of noise in the data. To assess the margin of error in the candidate pool size, the sensitivity of candidate pools to uncertainties in energy calculations was explored. Candidate pools thus far had been derived via hulls constructed from the exact calculated energies for each crystal structure. We implemented a method, inspired by the probabilistic hull sampling approach implemented in the original GCH method,^21^ where we recalculated the GCH and candidate pools many times, each time with random noise added to the energy of each structure. This noise was sampled from a uniform distribution, centered about 0, with a standard deviation equal to the estimated random error in the energy calculation. Across many iterations, this gives an indication of the spread of calculated candidate pools when accounting for uncertainty in the calculated energies. We estimated the random (nonsystematic) errors as 1.3 kJ/mol for the force field based CSP set for TTBI; 1.0 kJ/mol for the dispersion-corrected density functional theory (DFT) CSP landscape of galunisertib and 0.4 kJ/mol for the monomer-corrected DFT CSP landscape for ROY. (Discussion of these error estimates is provided in SI Section 7)

Figure 4 shows the distribution of the resulting candidate pool sizes for GCH runs using both adapted and average kernels using a 4 Å SOAP cutoff. For each case, the hull and pool sizes were calculated over 250 iterations. This value was found to be sufficient for the spread of candidate pools and the median candidate pool to remain as consistent as feasible across differing runs (SI Section 6). The results are further corroborated by the analogous data sets for kernels employing higher SOAP cutoff radii, which displayed similar behavior (SI Section 8).

**Figure 4:**
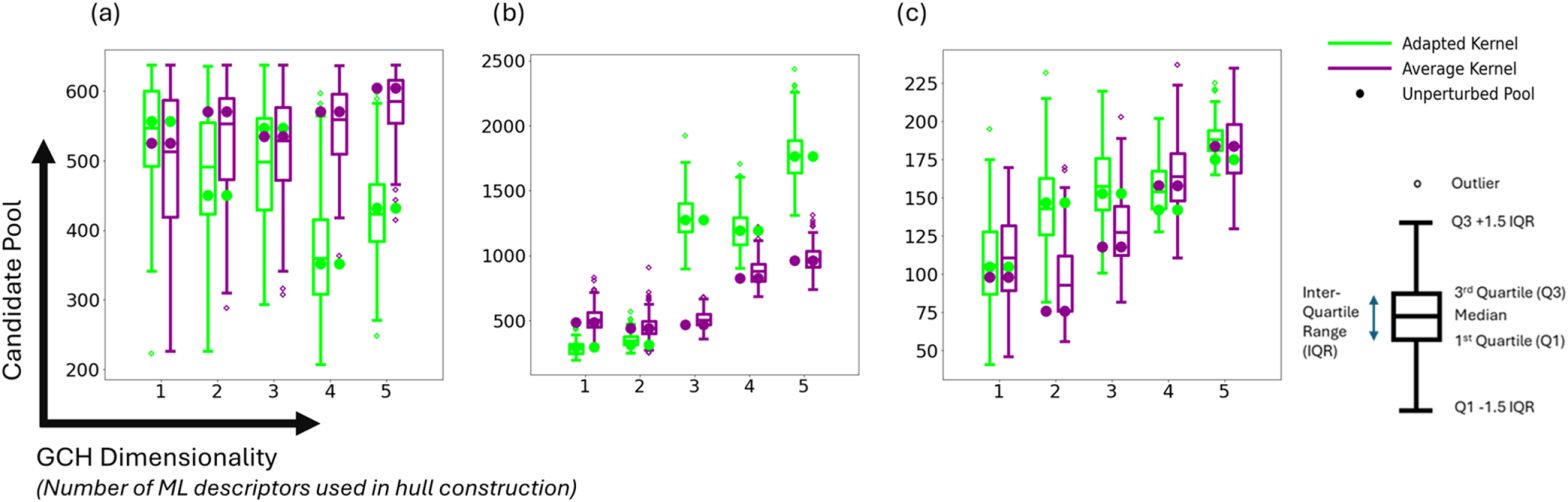
Box plots showing the spread of calculated candidate pools arising from the finalized iterative workflow for each system investigated from the average (purple) and adapted (green) kernel GCH implementations (4 Å cutoff) using different hull dimensionalities. Data is shown for the prediction sets of (a) galunisertib, (b) TTBI, and (c) ROY.

The results demonstrate that the differences in candidate pools due to the different kernels and choice of GCH dimensionality largely fall within the uncertainties in the candidate pools due to estimated uncertainties in the calculated energies. However, according to the Mann–Whitney U test (SI Section 9), many of the kernel comparisons still demonstrate statistical significance, indicating that one kernel filters candidates more efficiently in the corresponding case. Therefore, the performance differences are unlikely to be the result of random errors in energy calculations. We find examples where either kernel construction produces the smaller candidate pools; the relative kernel performance is dependent upon system, hull dimensionality and SOAP cutoff. For example, in the case of galunisertib, when using a 4 Å cutoff, the average kernel significantly outperforms the adapted kernel when using a 1D hull (*P*-value: 6 × 10^–5^) but the reverse is true (*P*-value: 1 × 10^–8^) when using a 2D hull. We conclude that the impact of the kernel construction upon the candidate pool size is at this point inconclusive and, disappointingly, does not demonstrate an improvement after adapting the kernel for molecular crystals.

### Interpreting Descriptors

Relationships between the ML descriptors derived from the GCH and more intuitive structural features were initially investigated visually, through CSP landscape plots of ML descriptors against intuitive descriptors that could be expected to be important to the system. Relationships that were identified in this way are summarized in Table 2 and an example of each relationship is shown in Figure 5.

**Figure 5:**
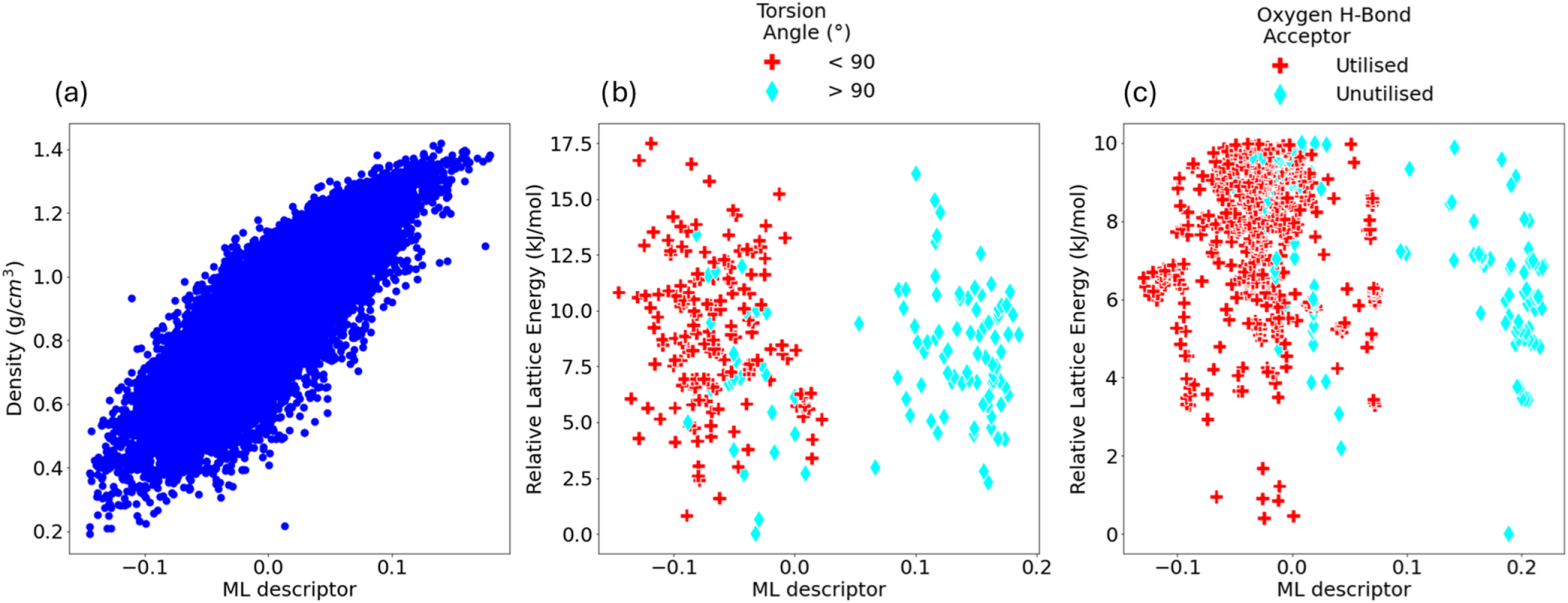
Examples of relationships identified between ML descriptors and intuitive structural features within CSP data sets. (a) Plot of density and an ML descriptor for crystal structures of TTBI. (b) Plot of ML descriptor vs relative lattice energy of ROY crystal structures colored according to the value of the key intramolecular torsion angle (Figure 7 a). (c) Plot of ML descriptor vs relative lattice energy of galunisertib crystal structures colored according to whether the oxygen atom (Figure 7 b) is involved in intermolecular hydrogen bonding in the crystal structure. In all cases, the ML descriptors here are those top ranked by a kPCA decomposition using an adapted SOAP similarity kernel with a 4 Å cutoff.

**Table 2:** Explored Systems and the Intuitive Features That Were Found to Relate to the ML Descriptors for the Corresponding Structure Sets

System	Related Intuitive Feature
DAP, TTBI, Trimesic Acid	Density
ROY	Intramolecular Angle
Galunisertib	Hydrogen Bonding

DAP, TTBI, and trimesic acid are all systems with porous polymorphs, for which density is a property of clear relevance and with relation to possible experimental constraints applied in synthesis: pressure or solvent inclusion can stabilize high or low-density structures, respectively.^1^ The intramolecular dihedral angle between rings in ROY has been shown to relate to the color of the crystal^31^ and is, therefore, a useful descriptor that relates to crystal properties. Molecular conformation is also a property that can be impacted by experimental constraints. For example, solvent choice can influence the populations of conformations present in solution,^33^ impacting nucleation rates. For galunisertib, hydrogen bonding would be expected to be a relevant descriptor due to the multiple possible motifs.^7^ Hydrogen bonding is also an important structural feature of molecular crystals that can influence properties such as stability, solubility, and mechanical properties.^34^ The visual relationships shown in Figure 5b,c do indicate clear relationships—the separation of data points along the machine-learned descriptors appearing to relate to the classification given. However, the relationships are imperfect—with “overlapping classes” in some regions of the plots. This could not clearly be assigned to any difficult region of the data. For instance, it did not appear to be the case that points in the overlapping classes of Figure 5b corresponded to conformations close to 90°. However, use of visualizations incorporating additional high-ranked machine-learned descriptors did indicate that inclusion of these secondary descriptors may partially aid separation of classes (see SI Section 11).

Exploring density as an intuitive descriptor, we can recall our tests on the utility of convex hulls constructed upon landscapes of energy and density in identifying stabilizable structures. In some cases where density was expected to be a key descriptor—e.g., TTBI—this did prove advantageous. Having identified molecular conformation to be a key intuitive descriptor in the case of ROY—via its relation to the highest ranked ML descriptor—we then tested whether descriptors based on torsion angle could also be used in the construction of effective convex hulls (SI Section 10). Results were generally on-par with application of the GCH. This may mean that in some instances effective convex hulls could be constructed using energy and intuitive descriptors alone. However, this is reliant upon the intuitive descriptor chosen by a researcher being one that proves effective and optimal—which is not guaranteed. The GCH, however, pairs this performance when using ML descriptors that have been interpreted with the additional benefit of still presenting sets of likely stabilizable structures even if the interpretations of the descriptors and coupled constraints would not be anticipated, or are complex—such as relating to multiple intuitive descriptors.

The primary focus of our investigations of the relationships between ML descriptors and intuitive structural features was in demonstrating the potential of the GCH—using different kernels—in deriving meaningful descriptors and in recovering patterns that could be identified by expert knowledge, as this could be seen to reflect sensible kernel construction. Visual comparisons suggested that ML descriptors derived via the adapted kernel may be more interpretable, i.e., relate more clearly to intuitive descriptors, than those derived from the average kernel (SI Section 12). For a more rigorous comparison between kernels, we sought to quantify these relationships.

The correlation with crystal density was observed to be approximately linear, so the strength of the relationship was quantified using *R* ^2^ calculated for a linear regression of density against the ML descriptor. In each case, *R* ^2^ was calculated for density with each of the first 32 kPCA components and we report the strongest correlation (see Table 3 for results using a 4 Å SOAP cutoff). These comparisons show that density is usually most strongly correlated with the first kPCA component (i.e., the kernel eigenvector with the largest eigenvalue) and the relationship for the adapted kernel is stronger or on-par with the result from the average kernel.

**Table 3:** *R* ^2^ Values for the Best ML Descriptor–Density Linear Relationships Identified for the DAP, TTBI, and Trimesic Acid Systems Using Each Kernel Type^a^

System	Kernel Type	*R* ^2^	Best Component
DAP	Adapted	0.697	1
	Average	0.632	1
TTBI	Adapted	0.697	1
	Average	0.698	1
Trimesic Acid	Adapted	0.564	2
	Average	0.359	1

a All kernel constructions used a 4 Å SOAP cutoff. The final column shows the kPCA component that most strongly correlates with crystal density (component 1 is the kPCA component corresponding to the highest kPCA eigenvalue).

The dependence of this relationship with density on the SOAP cutoff distance was investigated for the DAP structure set (Table 4). We observe that the correlation increases with cutoff distance up to 8 Å for both kernel constructions, which is unsurprising as density is a long-range structural feature, further demonstrating the potential of the GCH approach in deriving and implementing theoretically sensible structural descriptors.

**Table 4:** Linear Regression *R* ^2^ Values for the Best ML Descriptor–Density Relationships Identified for the DAP Systems Using Each Kernel Type and Various SOAP Cutoff Radii^a^

Kernel Type	SOAP cutoff (Å)	*R* ^2^	Best Component
Adapted	4	0.697	1
	6	0.757	1
	8	0.883	1
	10	0.797	1
Average	4	0.632	1
	6	0.759	1
	8	0.882	1
	10	0.800	1

a The final column shows the kPCA component that most strongly correlates with crystal density (component 1 is the kPCA component corresponding to the highest kPCA eigenvalue).

For the case of the adapted kernel, the correlations are shown in Figure 6, showing the strengthening of the relationship with increasing cutoff (albeit with a nonlinear character to the relationship). Although the difference in the correlation for the average and adapted kernels disappears at larger SOAP cutoff distances, the stronger relationships shown for the 4 Å cutoffs demonstrates an advantage of the adapted kernel in recovering meaningful relationships of the ML descriptors to density.

**Figure 6:**
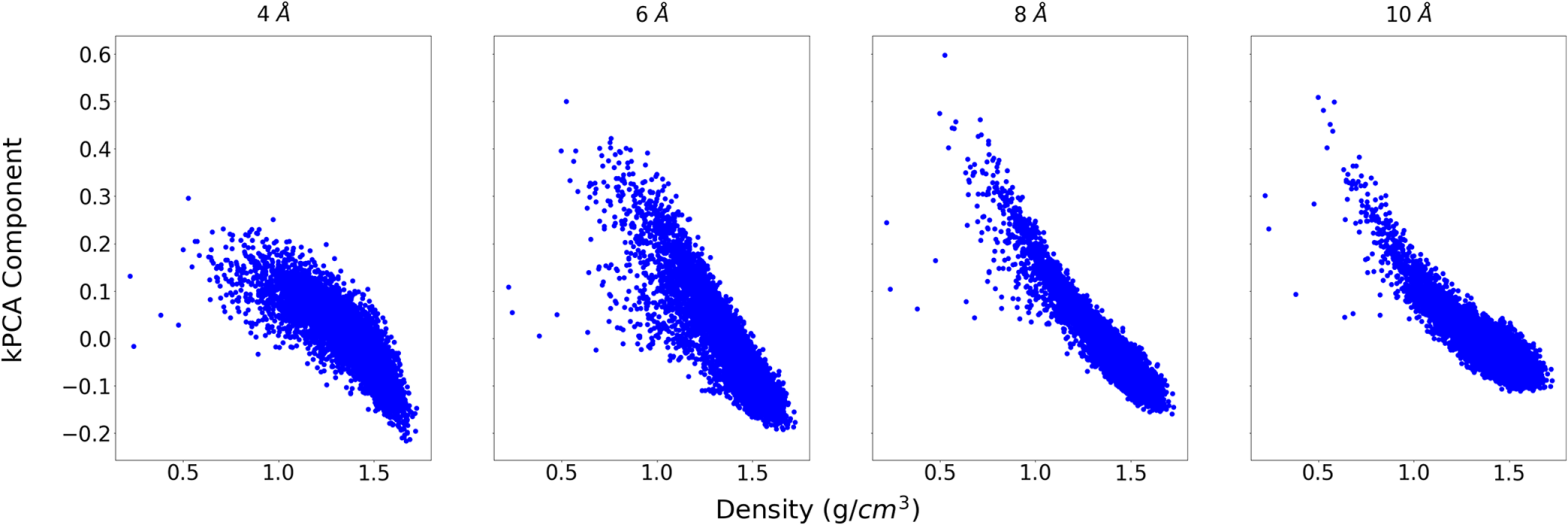
Plots of the top-ranked ML descriptors of DAP (from the adapted kernel constructions with varying SOAP cutoffs) against the crystal density.

To investigate the strength of categorical relationships—conformations of ROY and hydrogen bonding in galunisertib—we employed supervised machine learning. We measured the accuracy of Support Vector Classification, as implemented in sklearn,^35^ in predicting the classification of each crystal structure from the value of the machine-learned descriptor(s) for that structure. Higher predictive accuracy implies a clearer relationship between machine-learned and intuitive descriptors.

The intuitive descriptors were assigned to structures so as to formulate investigation of the relationships as a binary classification problem. Each ROY structure was assigned to class 0 or 1 according to whether the absolute value of the intramolecular C–N–CS angle (Figure 7a) was greater than or less than 90 deg (the average angle for molecules in the asymmetric unit was taken for *Z*′ > 1 crystal structures). Each galunisertib structure was assigned to class 0 or 1 according to whether or not the oxygen atom (Figure 7b) acts as an intermolecular hydrogen bond acceptor within the crystal structure. Hydrogen bonding was determined using the motif search in Mercury^36^ (SI Section 13).

**Figure 7:**
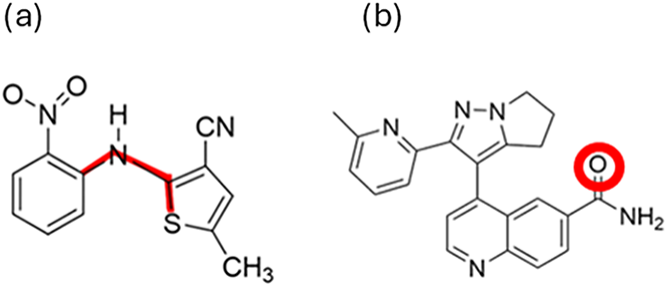
Torsional angle in the ROY molecule (a) and oxygen hydrogen bond acceptor in galunisertib molecule (b) used in defining the intuitive descriptors of the respective crystal structure sets.

Figure 8 shows the balanced accuracy scores of a linear support vector machine in learning the class of a crystal structure from its machine-learned descriptor value(s) as derived from various kernels. The assessment was performed for each case, learning from:

1. A single machine-learned descriptor. The results shown are that of the descriptor that resulted in the highest accuracy for each case across, individually assessing the first 32 kPCA components.
2. A combination of the first five machine-learned descriptors. All subsets/combinations of the five top-ranked kPCA components were tested and the accuracy shown is that from the combination that resulted in the highest accuracy for each case.

In each case, we also assessed the influence of the weighting, *C*, assigned to the hinge loss when training the classifier.

**Figure 8:**
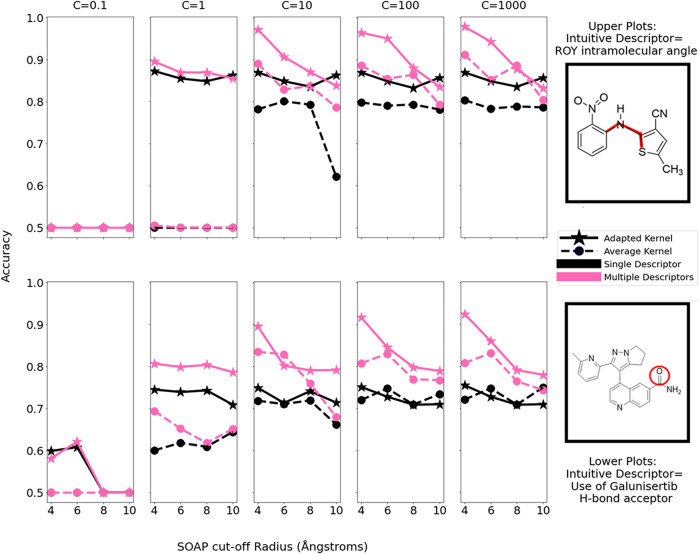
Plots showing the balanced accuracy of SVC models in learning the values of an intuitive descriptor from either single (black) or multiple (pink) ML descriptors as a function of the underlying SOAP cutoff radii. Line style denotes the type of kernel construction used to derive the ML descriptors.

These results show a general trend of greater learning performance in cases where the machine-learned descriptors have been derived via the adapted kernel. This trend is maintained across varying model parameters and SOAP cutoff radii. For both systems, balanced accuracies above 0.9 are achieved with the adapted kernel when using multiple ML descriptors as input to the classifier. The results demonstrate that the kernel adaptations lead to clearer relationships between machine-learned and intuitive descriptors and therefore more chemically meaningful machine-learned descriptors. The improved performance when learning from multiple descriptors reinforces the earlier suggestion that inclusion of secondary descriptors could aid the separation of intuitive classes.

### Energy Prediction

As a third test, we assessed the utility of the different kernel constructions within Gaussian Process Regression (GPR) in predicting energies of molecular crystal structures.

GPR models were trained to predict the relative total energies of *Z*′ = 1 chlorpropamide crystal structures learning from their similarity, as determined by the kernel to be tested, to training set samples. Crystal structures were taken from a previous CSP study,^17^ which used a bespoke workflow to handle the molecular flexibility during optimization, using a pairwise interatomic force field FIT^37,38^ and permanent electrostatics from atomic multipoles for intermolecular interactions combined with a DFT-based model of intramolecular energy. The target energies for GPR were calculated from single-point energy calculations using periodic DFT with the PBE functional, GD3BJ dispersion correction and 500 eV basis set cutoff, using the Vienna Ab Initio Simulation Package (VASP).^39–41^

An adaptation of the sklearn^35^ *Gaussian Process Regressor* class^42^ allows implementation of a precomputed kernel. Using this class, we establish a GPR workflow in which the SOAP kernel matrix for the training set both provides the training data and acts as the kernel used to determine the prior.

After preprocessing of the initial structure set (∼16,000 crystal structures) to remove potential unphysical structures based on a total energy cutoff criterion (SI Section 14), a 2000 member test set was randomly selected. The remaining valid structures were split at random into 500 member training set blocks.

For each kernel to be tested, a suitable total training set size was determined via an iterative training process:

1. For a 500 member initial training block, the relevant kernel subsets are extracted to obtain the GPR kernel, the training feature data, and the test feature data;
2. Absolute total energies for the training block were calculated;
3. The target data (relative total energies) was calculated from the absolute total energy data for each structure by setting the energies relative to the global minimum across the training set used so far
7 $$E_{x}^{\mathrm{Rel}}=E_{x}-\min\left\{E_{i}|i\in \mathrm{train}\right\}$$
4. The model performance was evaluated using Mean Average Error (MAE) and Root Mean Square Error (RMSE)
5. The training set was increased by addition of another 500 member training block to the overall training set and steps 1–4 were repeated using the expanded training set;
6. This expansion of the training was repeated until it was determined that the identified qualitative trends would not change and that further training would not justify the computational cost.

This testing continued up to a training set of 6000 structures. The resulting learning curves are shown in Figure 9. It was deemed that further investigation via additional single-point calculations and model training would not justify the computational cost as the training curves for the adapted kernel had reached satisfactory errors and the training curves for the average kernel—particularly using a 6 Å cutoff—could not be reliably expected to converge. Moreover, the shape of the training curves using the average kernel suggest a mismatch between the kernel measure of similarity and structural features that correlate with lattice energy. The performance gap between kernel implementations was assumed to be such that further training would not reverse the qualitative trends seen, which showed that GPR implementing the adapted kernel learned relative total energies more quickly.

**Figure 9:**
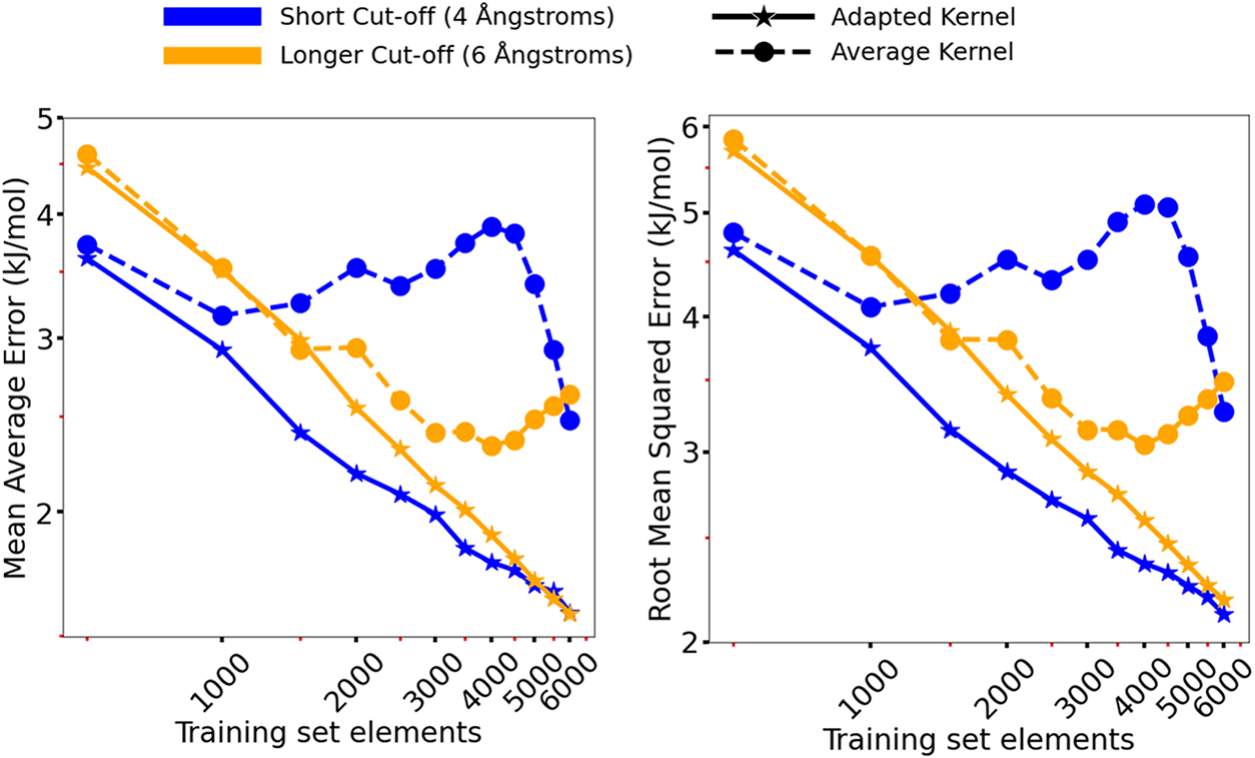
Average RMSE and MAE of energy predictions for the extended case of the chlorpropamide system as a function of training set size. Line and marker style denote the kernel construction used in the GPR model. Different colors indicate different cutoff radii of the underlying SOAP descriptors used to derive the kernel.

To investigate the unusual learning performance of the average kernels, parity plots for the test set predictions at a few key points in the training were generated (SI Section 15). These plots showed no particular outliers in the predictions—suggesting that, for example, increasing mean average errors when increasing training set size for the average kernel with the 4 Å cutoff were indeed representative of a general degradation in performance. We assumed that this is due to the behavior of the corresponding models when encountering particular (sets of) training structures.

After the initial progressive testing, the test and training data was then merged and 5-fold cross-validation was applied using all 8000 structures for which energy data was available—corresponding to 6400/1600 structures in the training/test set folds respectively. At each step of cross-validation, the model was freshly trained on the training set-fold, with the energies to be learned being set relative to the global minimum for that fold. The mean and standard deviation of the RMSE and MAE over the cross-validation are reported in Table 5.

**Table 5:** Average RMSE and MAE Values of Energy Prediction on the Extended Chlorpropamide Set, Measured via 5-Fold Cross-Validation upon the Entire Set of 8000 Structures for Which Periodic DFT Single-Point Energies Were Calculated^a^

Kernel Type	Cutoff (Å)	RMSE (kJ/mol)	MAE (kJ/mol)
Average	4.0	3.056 ± 0.073	2.309 ± 0.050
	6.0	3.659 ± 0.138	2.764 ± 0.133
Adapted	4.0	2.168 ± 0.036	1.638 ± 0.032
	6.0	2.123 ± 0.034	1.542 ± 0.054

a Results are shown for both kernel constructions and two cutoff radii.

The results indicate a clear, 29 to 44% (based on MAE), improvement of the adapted kernel over the average kernel with regard to energy prediction. This is seen at both 4 and 6 Å underlying cutoff radii. The difference between kernels is much greater than the standard deviations on the calculated errors across folds (Figure 10), indicating a significant performance gap.

**Figure 10:**
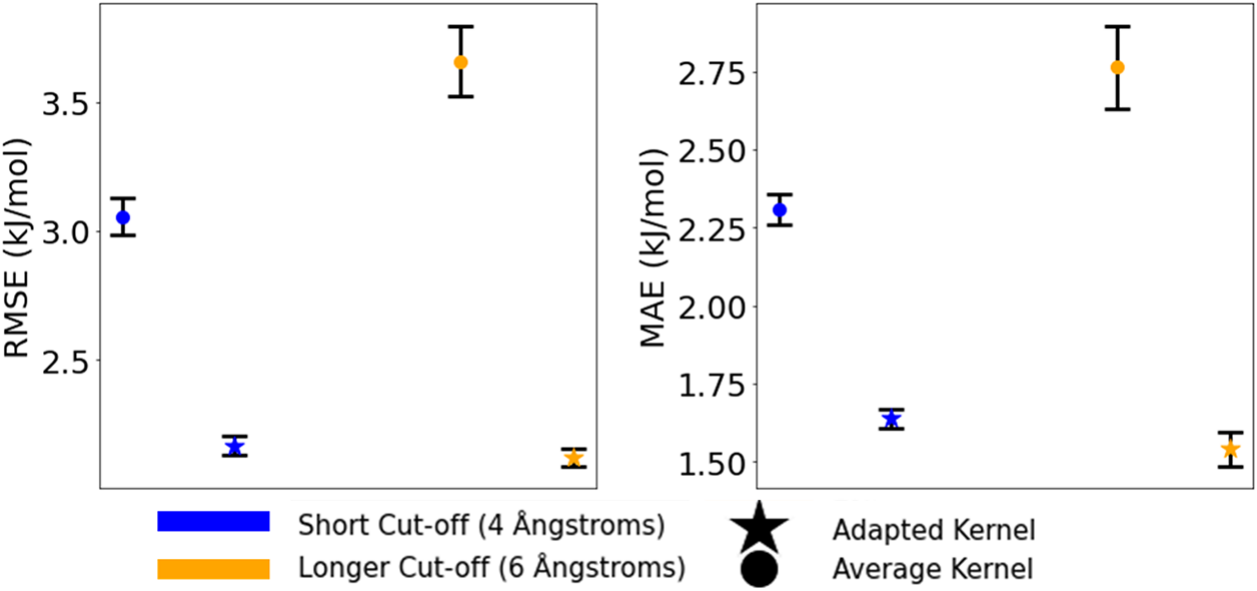
Average RMSE and MAE values of energy prediction on the chlorpropamide CSP set, measured via 5-fold cross-validation using the 8000 crystal structures for which DFT single-point energies were calculated. Color denotes the SOAP cutoff radius and marker style indicates the kernel construction used in the GPR model. The error bars about each point correspond to one standard deviation of errors measured in cross-validation.

GPR using the adapted kernel approached an MAE of 1.5 kJ/mol and achieved errors below 2.0 kJ/mol with just 3500 structures in the training set (Figure 9). This error is similar to typical polymorph pair lattice energy differences.^16^ However, the required training set size to reach satisfactory errors is too large to be cost-effective during CSP. Therefore, while neither implementation would be ideal for energy prediction in this instance, the adapted kernel implementation has shown initial promise over the average kernel implementation—which failed to reach satisfactory errors even with 6400 training structures.

However, real-world energy prediction often focuses upon a low-energy region of the landscape and performance across this narrower region may differ from the testing discussed above. Learning performance across lower-energy subsets of the full landscape was therefore subsequently tested by extracting structures within varying energy windows and retraining/evaluating the model. 5-fold cross-validation was performed in each case. Table 6 shows the energy windows investigated, the corresponding training set sizes, and the learning performance for each kernel implementation.

**Table 6:** Average RMSE and MAE Values of Energy Prediction on the Chlorpropamide CSP Structures, Measured via Cross-Validation upon Given Low-Energy Subsets of the Entire Set of 8000 Structures for Which Periodic DFT Single-Point Energies Were Calculated^a^

			4 Å cutoff		6 Å cutoff	
Energy Window (kJ/mol)	Train Set Size	Kernel Type	RMSE (kJ/mol)	MAE (kJ/mol)	RMSE (kJ/mol)	MAE (kJ/mol)
50	5950	Average	3.213 ± 0.062	2.429 ± 0.045	3.335 ± 0.102	2.511 ± 0.070
		Adapted	2.075 ± 0.014	1.569 ± 0.008	2.013 ± 0.023	1.484 ± 0.018
40	4648	Average	4.169 ± 0.173	3.149 ± 0.146	2.784 ± 0.108	2.072 ± 0.101
		Adapted	2.00 ± 0.039	1.505 ± 0.030	2.023 ± 0.041	1.490 ± 0.031
30	2312	Average	3.056 ± 0.121	2.274 ± 0.073	2.393 ± 0.054	1.780 ± 0.046
		Adapted	1.940 ± 0.034	1.459 ± 0.018	2.069 ± 0.089	1.509 ± 0.047
20	659	Average	2.396 ± 0.059	1.759 ± 0.047	2.246 ± 0.128	1.694 ± 0.098
		Adapted	1.980 ± 0.246	1.476 ± 0.184	2.015 ± 0.186	1.507 ± 0.119

a These results are shown for each tested underlying descriptor cutoff radius and kernel construction used in the GPR model.

These findings suggest that energy prediction with reasonable errors may be possible with feasibly small training sets for both implementations, if within a limited domain. Under these circumstances, the adapted kernel continues to display an advantage over the average kernel—though the performance gap is significantly narrower than when assessed over the entire crystal energy landscape. The prediction errors for lower energy subsets are visualized in SI Section 16.

Overall, the GPR results demonstrate improved utility of the adapted kernel over the average in energy prediction—particularly when using larger structure sets or structure sets spanning a wider total energy range.

## Conclusions

We describe an adapted SOAP kernel for quantifying the similarity of molecular crystal structures, and its application in the Generalized Convex Hull for analyzing crystal structure prediction landscapes. The kernel adaptation limits comparisons of atom environments to analogous atoms, taking molecular symmetry into account where needed. We have assessed the impact of the changes to the kernel on the effectiveness of the GCH, the interpretability of the derived descriptors, and the performance of the kernel in energy prediction.

Overall, our results demonstrate that the choice of kernel used does impact the analysis of landscapes of molecular crystals, and the impact of adapting the kernel construction to molecular crystals differs for the different applications investigated here.

Studies of candidate pool selection demonstrated that, for molecules where some polymorphs result from desolvation of solvates (galunisertib and TTBI), the GCH can highlight smaller CSP structure sets than a traditional lattice energy cutoff. The results demonstrate the value of the GCH in identifying synthesizable, high-energy, kinetically trapped polymorphs. However, the effectiveness is inconsistent: for a third molecule, ROY, an energy cutoff produces consistently smaller candidate pools than the GCH. The candidate pools were also inconclusive in demonstrating improved results with the adapted kernel.

We do, on the other hand, find that the SOAP kernel that has been adapted for molecular crystals leads to more intuitively interpretable descriptors. The most important components from kPCA of the adapted SOAP kernel are found to relate to crystal density, molecular conformation and hydrogen bonding motif in the CSP structure sets of DAP, ROY and galunisertib, respectively. The adapted kernel described herein was also demonstrated to improve upon the average kernel in predicting DFT-level crystal energies via Gaussian process regression. This result suggests that the structural similarity measured by the adapted kernel relates more strongly to the structural features that influence lattice energy.

## Supplementary Material



## Data Availability

Kernel Generation scripts for both the adapted and average kernels will be available at: https://gitlab.com/jennie_martin/molecular-crystal-similarity. These scripts also cover GCH and machine-learned descriptor calculation. Structure files for predicted DAP structures and DFT single-point energies for chlorpropamide are available at: https://doi.org/10.5258/SOTON/D3722.

## Notes

Additional data plots and results; details of crystal structure prediction and known structure match identification; extended methodological details; results of statistical tests (PDF)
