# Supplementary material for: An Adapted Similarity Kernel and Generalized Convex Hull for Molecular Crystal Structure Prediction
Source: Cryst Growth Des. 2025 Oct 23;25(21):9461–74. doi: 10.1021/acs.cgd.5c01220 (PMC12593367; doi:10.1021/acs.cgd.5c01220)
Supplement: Supplementary file 1 [file cg5c01220_si_001.pdf]

An Adapted Similarity Kernel and Generalized Convex Hull for Molecular Crystal Structure Prediction

Supporting Information

Jennie Martin, Michele Ceriotti, Graeme Day

October 15, 2025

Contents

1 Selection of Predicted Structures Corresponding to Known Polymorphs 3

2 Crystal Structure Prediction Methodology: DAP 5

3 Explanations of Polymorphs Missing from Datasets 7

4 Full CSP Landscapes 8

5 Rankings and Correlations of Rankings of Known Structures 9

6 The Number of Iterations for Perturbed Hulls 11

7 Random Error Estimates for Perturbed Hulls 12

8 Perturbed Hull Candidate Pools at Higher Cut-Off Radii 13

9 Statistical Significance Tests 15

10 Energy-Torsion Angle Convex Hulls for the ROY System 17

11 Additional Plots for Interpretation of Machine-Learned Descriptors 19

12 Visual Comparisons of ML-Intuitive Descriptor Relationship Strength 24

13 Hydrogen Bonding Motif Search 29

14 Removal of Unphysical Structures 30

15 Parity Plots for Energy Predictions **31**

16 Graphs of Errors in Machine Learning of Energies with Smaller Training Sets **32**

References **34**

1 Selection of Predicted Structures Corresponding to Known Polymorphs

Candidate pools were determined based on the windows necessary to ‘capture all known polymorphs’. This was required identification of structures within the CSP sets that correspond to the known polymorphs.

For the TTBI system, we used the structures identified in reference [1] as matches to the experimental polymorphs.

For the ROY system, reference [2] identified the Z’=1 structures corresponding to the known Z’=1 polymorphs, and supplemented the dataset with the known experimental structures of three Z’=2 polymorphs. We used these identified structures in determination of the candidate pool, alongside the 24th ranked structure - which was identified as being a match to the newest ROY polymorph, O22 [3].

For the galunisertib system, we performed searches using the CrystalPackingSimilarity functionality in the CSD API [4], inspired by the COMPACK [5] algorithm. We identified as matches to a given known polymorph any structure for which 30 molecule clusters of the respective crystal structures could be successfully overlaid. We used the minimal tolerances required to identify a match in each case - which ranged from 0.2 Å/20° to 0.3 Å/30° and selected the lowest energy match in each case. On the basis of these searches we determined the candidate pools - relying upon the correspondence between predicted and known structures shown in Table S1 These selections are mostly the same as the match selections identified in reference [6], with the exception of Form V, for which we identified a lower energy match within the set. It is important to note that the structure set did not contain a match to Form I, and so that polymorph was not considered when calculating candidate pools.

Form	Rank of Match (Corresponds to Filename)
II	366
III	543
IV	5
V	64
VI	47
VII	2
VIII	39
IX	6
X	13

Table. S1: Energetic rankings, within the literature structure set [6], of the structures corresponding to known polymorphs of galunisertib. Structures in the original dataset are also labeled according to this ranking.

2 Crystal Structure Prediction Methodology: DAP

The CSP landscape of DAP used in this work was generated using a previously developed quasi-random workflow [7], for which the code is now available [8]. The landscape used here is the result of a 'rigid-molecule' CSP run. Trial crystal structures of the gas-phase conformer of the molecule (As optimized at DFT level in Gaussian09 [9] using PBE0+GD3BJ/6-311G**) were generated and then optimised using pairwise interatomic force field FIT [10, 11] alongside permanent electrostatic contributions from distributed atomic multipoles up to rank hexadecapole. Multipoles were calculated at the same level of theory as gas-phase conformer optimisation.

Quasi-random searching continued until obtaining 10, 000 successfully optimised Z'=1 crystal structures in each of the 25 most common space groups for organic systems [12] ($P12_1/c1$,$P2_12_12_1$,$P\bar{1}$, $P12_11$,$Pbca$,$C12/c1$,$Pna2_1$,$C121$,$P1$,$Pbcn$,$P1c1$,$P2_12_12$,$Fdd2$,$Pccn$,$P12/c1$,$I4_1/a$,$R\bar{3}$,$P4_1$, $P4_32_12$,$P4_12_12$,$P4_3$,$P3_2$,$P3_1$)

To restrict the search problem, as CSP was conducted for the purposes of data generation rather than true discovery, the search used only a single tautomer of DAP, the tautomer present in both fully characterized HOF structures [13] (Figure S1).

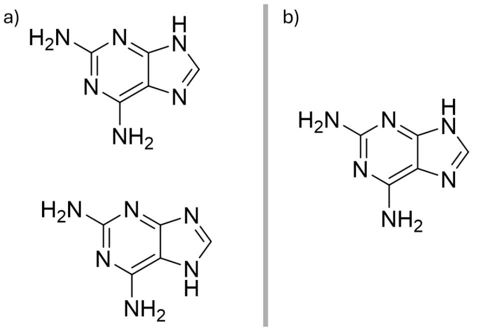

Fig. S1: Possible tautomers of DAP (a) and he tautomer used for CSP work, being present in both experimental HOF structures (b)

The landscape was searched only for matches to the known HOF-1 ([13]) structure, porous structures being of interest to us at the time and the HOF-2 structure being known to be Z'=3 [13] and so not possible to be predicted by the Z'=1 search. Matches were identified using the COMPACK [5] algorithm, as implemented in the CSD API [4], accepting as a match any crystal structure for which a 30/30 molecules of 30 molecule cluster could be overlaid with a 30 molecule cluster of the experimental crystal structure, to within tolerances of 0.2 Å and 20 °.

Table S2 shows the lowest lattice-energy match found:

Landscape	No. Unique Structures	Energetic Ranking of Match	Relative Lattice Energy (kJ/mol)	$RMSD_{30}$(Å)
Z'=1 (HOF 1 Search)	5825	2	3.905	0.316

Table. S2: Structure sets sizes and experimental match results from the Z'=1 landscape calculated in crystal structure prediction of DAP

3 Explanations of Polymorphs Missing from Datasets

Three of the CSP datasets used in this work are incomplete, in that they do not contain all known polymorphs. These sets are the DAP, galunisertib, and chlorpropamide systems.

In the case of DAP, as discussed in S.I Section 2, the dataset used in this work results from a Z'=1 CSP search, as such the Z'=3 DAP-HOF-2 form [13] is not present. There exists also a fully characterised Z'=1 anhydrate form. Given the initial purpose of the dataset as an exploration of porous structures, the landscape was not explored to confirm the presence of this non-porous form among predictions.

In the case of galunisertib, the dataset is taken from the literature [6]. This dataset does not contain a predicted structure corresponding to known form I of galunisertib. This may be because the datset provided considers only a low energy region of the full CSP landscape, and form I is known to be highly metastable. [6].

In the case of chlorpropamide [14], 8 of the 9 known polymorphs are found in the prediction set. The ninth (ε') polymorph, was likely missed due to the underlying molecular conformation not being sampled [14]. Whilst many of the known polymorphs were present in the dataset, the system was not considered as a promising system for the investigation of candidate pools due to concerns over the energy model used in derivation of the original dataset.

4 Full CSP Landscapes

It provides useful context for consideration of candidate pools to see the extent of full CSP landscapes. For example, the full CSP landscape of TTBI contains over 14,000 structures, and so many of the TTBI candidate pools in this work still represent a significant reduction of the dataset. Figure S2 shows the energy-density landscapes of the full available CSP landscapes for the systems of galunisertib, TTBI [1], and ROY. It should, however, be noted that the available landscapes of galunisertib [6] and ROY [2] used in this work and shown here are low-energy subsets of full CSP landscapes.

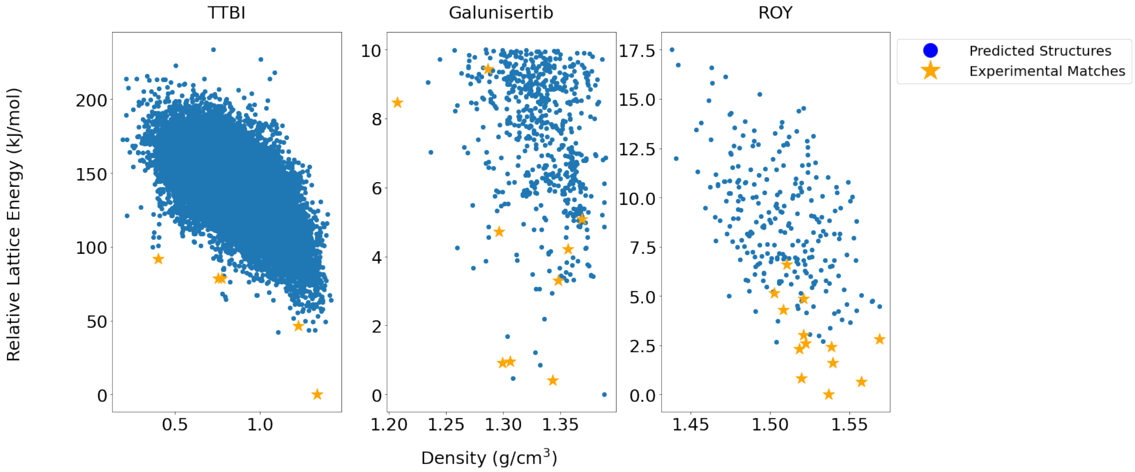

Fig. S2: Energy-density plots of the full available CSP landscapes of TTBI [1], galunisertib [6], and ROY [2], with predicted structures corresponding to known structures indicated.

5 Rankings and Correlations of Rankings of Known Structures

By comparing the rankings (as determined by purely by energies or determined by proximity to the GCH) of predictions corresponding to known structures, we can gain insight into the ‘order’ in which known structures are incorporated into a GCH candidate pool. That is, we can determine whether or not the most thermodynamically stable structures are more likely to lie close to the hull and so be recoverable within smaller candidate pools.

Table S3 displays the energetic rankings and GCH-based rankings of known structures for the galunisertib, TTBI, and ROY systems using 1D GCH constructions with a 4 Å cut-off. We calculated the kendall rank correlations between these energetic rankings and GCH rankings in each case. The results, shown in Table S4, indicate that there is some relationship (with significant correlations seen for the galunisertib and ROY systems) but this is not universal.

System	Energetic Rankings	Adapted GCH Rankings	Average GCH Rankings
Galunisertib	2,5,6,13,39, 47,64,366,543	1,11,1,73,72, 107,114,372,550	2,1,3,21,56, 11,74,1,521
TTBI	1,5,185,191,647	1,290,22,92,1	1,480,36,221,4
ROY	1,2,3,4,5,6,7, 10,12,23,24,32,37,72	1,3,1,6,1,7, 9,2,23,1,39,49,17,98	1,1,1,3,16,15,1, 5,7,20,46,11,63,91

Table. S3: The rankings of structures -within their respective prediction sets - corresponding to known polymorphs. Rankings are shown as determined by energetic ranking alone, and as determined by energy relative to the hull for GCH constructions. All GCH constructions used 1D hulls with a 4Å SOAP cut-off. For energetic rankings, a rank of 1 indicates the global minimum of the prediction set. For GCH-based rankings, all hull structures share the rank of 1.

Case	Coefficient	P-Value
Galunisertib - adapted	0.873	0.001
Galunisertib - average	0.479	0.075
TTBI - adapted	-0.105	0.801
TTBI - average	0.000	1.000
ROY - adapted	0.603	0.003
ROY - average	0.694	0.001

Table. S4: The calculated Kendall rank correlation coefficients, and corresponding p-values, for correlations between rankings of structures -within their respective prediction sets - corresponding to known polymorphs. These correlations have been calculated between rankings as determined by energy alone, and rankings as determined by energy relative to the hull for GCH constructions. All GCH constructions used 1D hulls with a 4Å SOAP cut-off.

6 The Number of Iterations for Perturbed Hulls

Given the random nature of the energy perturbations applied in this work, it was required to determine the number of iterations of the workflow necessary to obtain a consistent estimate of the spread of calculated candidate pools. A single example case (TTBI, adapted kernel, 4 Å cut-off, 1D hull) was used to test this. Figure S3 shows the spread of calculated candidate pools - represented as box plots, tested across different numbers of iterations of the loop. The workflow using each number of iterations was tested four separate times, to explore the consistency of results.

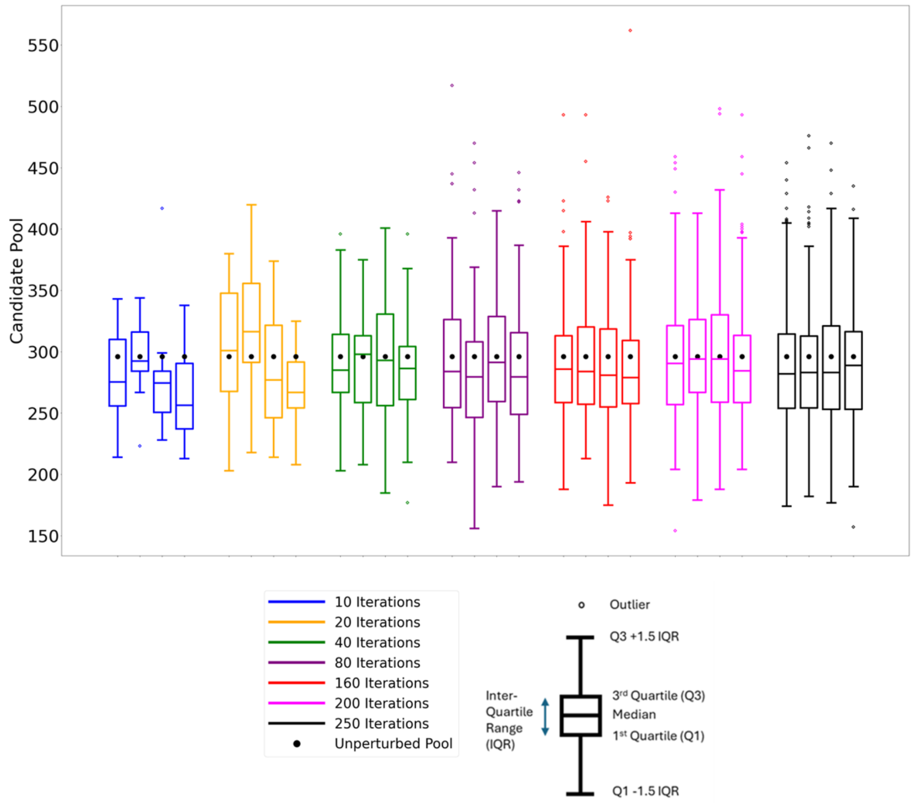

Fig. S3: Boxplots showing the spread of calculated candidate pools for a single example case (TTBI, adapted kernel, 4Å cut-off, 1D hull), when the iterative workflow to account for energetic uncertainty was applied. Each colour signifies a different number of iterations of the loop used and each boxplot of a given colour corresponds to a separate 'run' of the workflow with that many iterations.

Based upon the above results, it was determined that 250 iterations of the loop was sufficient to obtain measures of the spread of candidate pools that were not significantly impacted by the arbitrary nature of the specific random perturbations implemented.

7 Random Error Estimates for Perturbed Hulls

Given the specificity of the methods applied in the CSP procedures used for the various systems, clearly benchmarked non-systematic errors were not freely available for all systems. To attempt to gauge sensible estimates of these errors, different approaches were employed.

For the case of TTBI, the energies were calculated using intermolecular forcefield FIT [10, 11] with permanent electrostatic contributions from distributed atomic multipoles.[1]. A reliable benchmark [15] of this energy evaluation method is available - assessed on the X23 [16] set of experimental lattice energies. From this data, the non-systematic error was estimated by the difference in magnitude of the Mean Absolute Error (MAE) and the Mean Signed Error (MSE):

$$Err_{Rand} = MAE - |MSE| \tag{1}$$

This results in an approximate estimate of the non-systematic error of 1.3 kJ/mol.

For the case of ROY, the prediction study provided indication of the error of the method on the ROY dataset - providing a figure of 0.4 kJ/mol Root Mean Square Error (RMSE).[2]. As precise data from which to calculate the non-systematic error was not available, this value was utilised as-is having been taken to represent a top bound of the non-systematic error - which cannot exceed the 0.4 kJ/mol estimate.

For the case of Galunisertib, reliable error estimates for the energy evaluation method (PBE + Neumann Perrin Dispersion correction) [6] could not be found. The useful accuracy of the method can be expected to lie between that of the methods used for TTBI and ROY energy evaluation. As such, an estimate of 1.0 kJ/mol was used.

8 Perturbed Hull Candidate Pools at Higher Cut-Off Radii

Figures S4 and S5 show the spread of candidate pools when using perturbed energies and 6 and 8Å cut-off radii for the SOAP descriptors respectively. Note that kernel calculations for TTBI with SOAP cut-off radii > 4 were not performed. Sub-figures (b) therefore indicate the data for the ROY system and not the TTBI system.

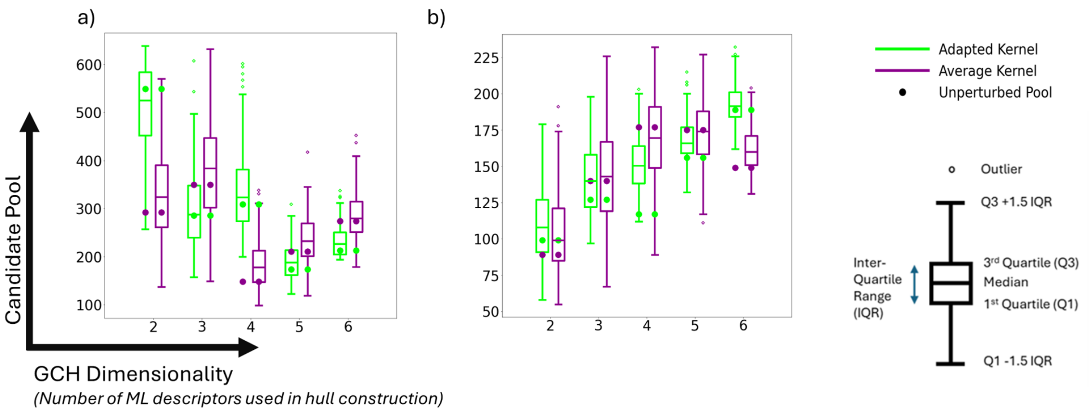

Fig. S4: Box plots showing the spread of calculated candidate pools arising from the finalised iterative workflow for each system investigated from the average and adapted kernel GCH implementations (6 Å cut-off) using different hull dimensionalities. Data is shown for the prediction sets of a) galunisertib, b) ROY.

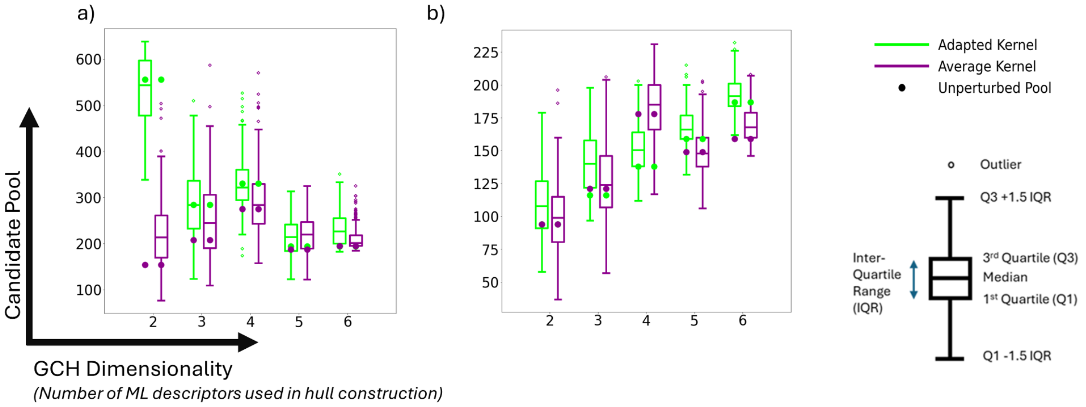

Fig. S5: Box plots showing the spread of calculated candidate pools arising from the finalised iterative workflow for each system investigated from the average and adapted kernel GCH implementations (8 Å cut-off) using different hull dimensionalities. Data is shown for the prediction sets of a) galunisertib, b) ROY.

9 Statistical Significance Tests

Tables S5, S6, and S7 show the significance of the differences in distributions of perturbed candidate pools for the systems of galunisertib, TTBI, and ROY respectively. Tests explored the significance of the differences in candidate pool distributions using different hull dimensionalities but all results refer to cases with a 4 Å SOAP cut-off. The significance test applied in each case was a one-tailed Mann Whitney U test, with the direction (i.e greater/lesser) being determined by the differences between the median values of the candidate pool distributions from adapted and average kernels. The test applied is named relative to the adapted kernel results, i.e ‘greater’ tests give the significance of the finding that the adapted kernel candidate pools skew higher than the average kernel candidate pools.

Hull Dimensionality	Test Applied	Statistic	P-value	Significant
1	greater	37474.5	5.832×10^{-5}	Yes
2	less	22219.5	1.134×10^{-8}	Yes
3	less	24829.5	3.528×10^{-5}	Yes
4	less	2028.5	1.921×10^{-73}	Yes
5	less	2428.5	1.663×10^{-71}	Yes

Table. S5: Results of Mann-Whitney U tests investigating the significance of the differences in the distributions of candidate pools using the average and adapted kernels for the case of galunisertib and a 4 Å cut-off (Fig 3a). The test applied is relative to the adapted kernel results, i.e ‘greater’ tests give the significance of the finding that the adapted candidate pools skew higher than the average.

Hull Dimensionality	Test Applied	Statistic	P-value	Significant
1	less	437.5	2.051×10^{-81}	Yes
2	less	7618.5	9.139×10^{-49}	Yes
3	greater	62500.0	1.114×10^{-83}	Yes
4	greater	60685.5	1.724×10^{-74}	Yes
5	greater	62499.0	1.128×10^{-83}	Yes

Table. S6: Results of Mann-Whitney U tests investigating the significance of the differences in the distributions of candidate pools using the average and adapted kernels for the case of TTBI and a 4 Å cut-off (Fig 3b). The test applied is relative to the adapted kernel results, i.e 'greater' tests give the significance of the finding that the adapted candidate pools skew higher than the average.

Hull Dimensionality	Test Applied	Statistic	P-value	Significant
1	less	29123.0	0.094	No
2	greater	56828.0	8.955×10^{-57}	Yes
3	greater	50593.0	2.392×10^{-33}	Yes
4	less	23870.0	2.449×10^{-6}	Yes
5	greater	35930.5	0.002	Yes

Table. S7: Results of Mann-Whitney U tests investigating the significance of the differences in the distributions of candidate pools using the average and adapted kernels for the case of ROY and a 4 Å cut-off (Fig 3c). The test applied is relative to the adapted kernel results, i.e 'greater' tests give the significance of the finding that the adapted candidate pools skew higher than the average.

10 Energy-Torsion Angle Convex Hulls for the ROY System

We measured two key torsional angles in the ROY molecule - in the in-crystal conformation - for each crystal. These two angles, τ_1 and τ_2 are shown in Figure S6.

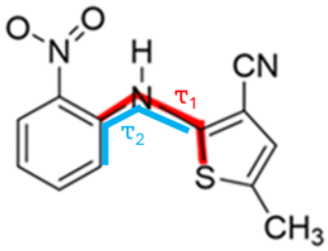

Fig. S6: Torsion angles τ_1 and τ_2 of the ROY molecule

We then aimed to constructed convex hulls of the ROY prediction set based upon energy and the torsional angles. We calculated the convex hulls and corresponding candidate pools for:

1. τ_1 vs Energy

2. τ_2 vs Energy

3. A 'two-dimensional' hull of τ_1 vs τ_2 vs Energy

In the single angle vs energy hulls, the absolute value of the angle was taken. In the case of the two-dimensional hull, the sign of the angles was adjusted such that τ_1 was always positive, and the sign of τ_2 was flipped if the sign of τ_1 was flipped. In order to construct a hull, a single value (of each torsional angle) was required for each crystal structure. Therefore, for the Z'=2 structures, the torsional angles were calculated separately (including determination of their sign) for each molecule in the asymmetric unit - before averaging across the resulting values for the asymmetric unit molecules.

The candidate pools are shown in Table S8:

Case	Pool
τ_1	101
τ_2	100
τ_1 and τ_2	113

Table. S8: Candidate pools for the ROY system as extracted from convex hulls on a landscape of energy and different torsion angles (τ_1 and/or τ_2)

11 Additional Plots for Interpretation of Machine-Learned Descriptors

Whilst the visualisations of ML-intuitive descriptor relationships show clear relationships, they still exhibit some overlapping distributions of classes.

It is therefore interesting to investigate whether any explanation for this limited separation can be identified. One key question is whether those points in the region of overlap for the ROY ML descriptor - intramolecular angle relationship (Fig 5b in the main text) lie close to the class boundary (i.e have angles close to 90°). Figure S7 displays the 1D GCH landscape (4 Å cut-off, adapted kernel) of ROY coloured by the absolute value of the intramolecular torsion angle. This data suggests that it is not the case that points in the overlapping region correspond to structures with an intramolecular torsion close to 90 °.

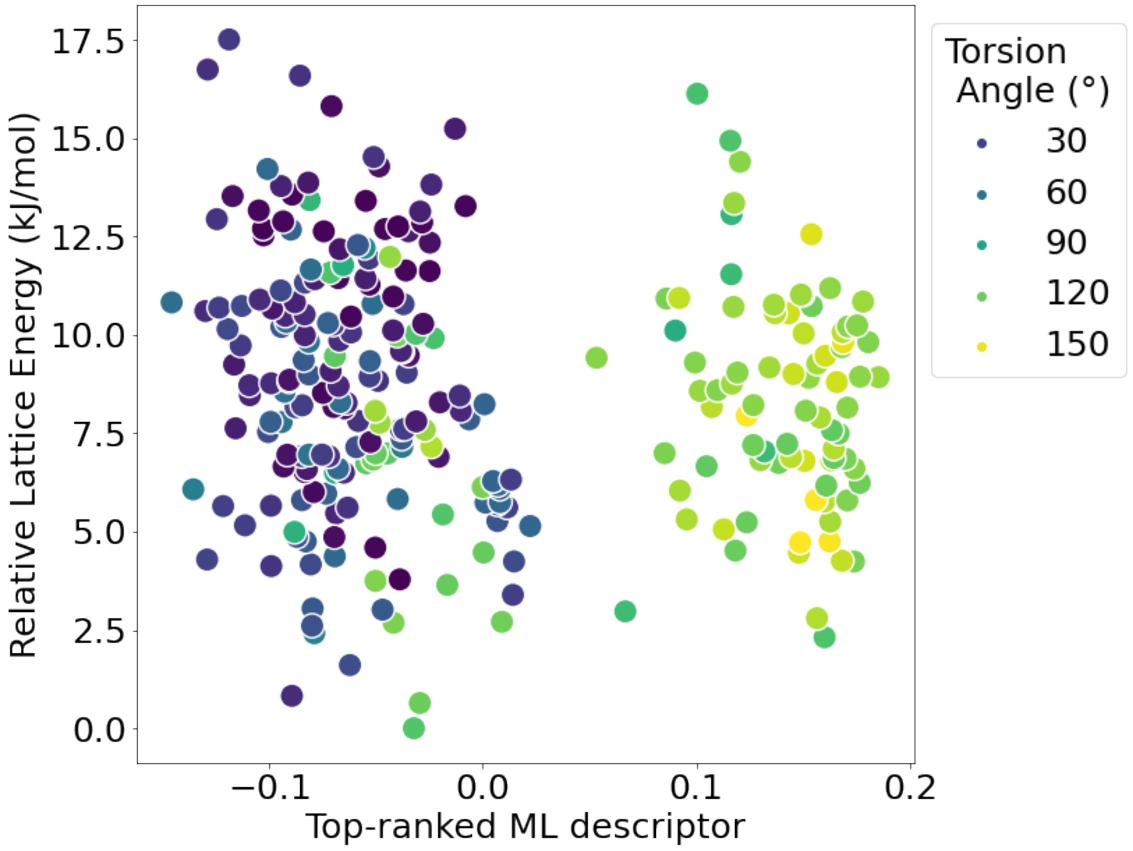

Fig. S7: 1D GCH landscapes (4 Å SOAP cut-off) of ROY from adapted kernel construction - coloured by the absolute value of a key intramolecular torsion in the underlying molecule. The ML descriptor plotted in is the kPCA component - of the top ranked five components in the eigenspectrum - that best related to the conformation classification - in this instance it is the top-ranked component

Another possibility to explore is that the distributions may be separable by considering secondary ML descriptors in conjunction with the top-ranked ML descriptors. This does appear to be the case, with incorporation of additional ML descriptors reducing , though not eliminating, the overlap of distributions of different classes (Figure S8,S9,S10, S11).

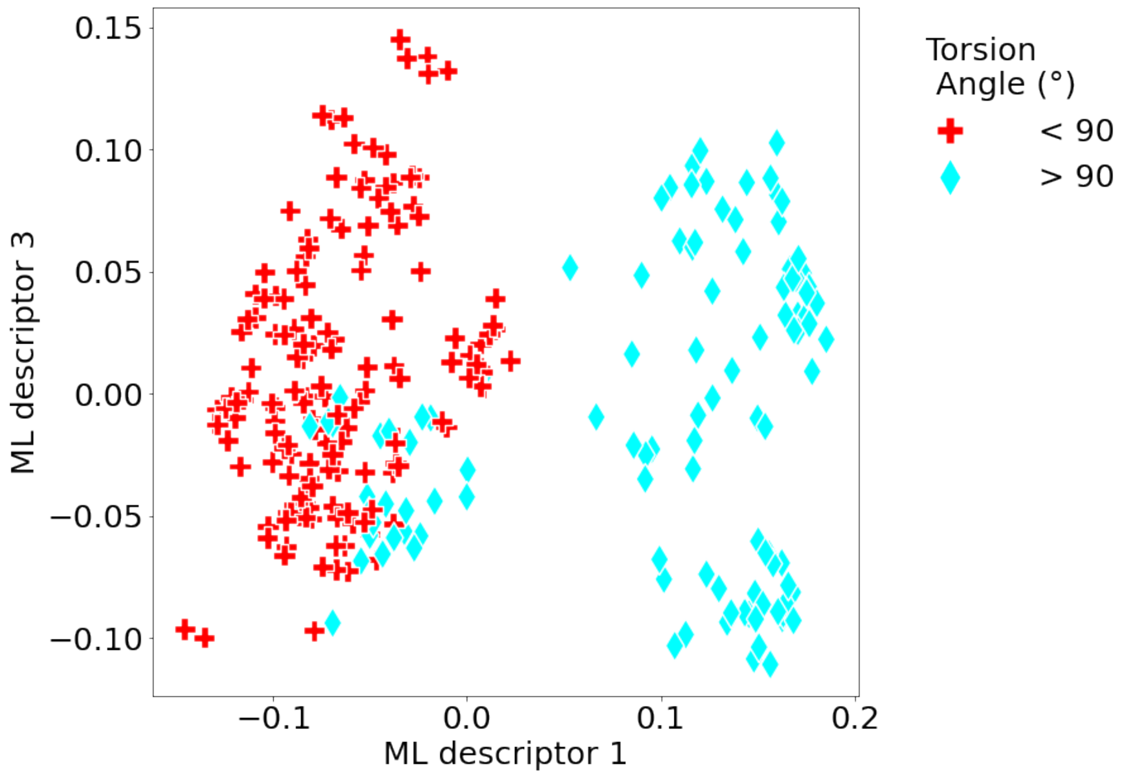

Fig. S8: ROY structure set plotted by the top two ranked ML descriptors from the adapted kernel construction (4 Å SOAP cut-off) - coloured by whether on not a key intramolecular torsion in the underlying molecule is acute

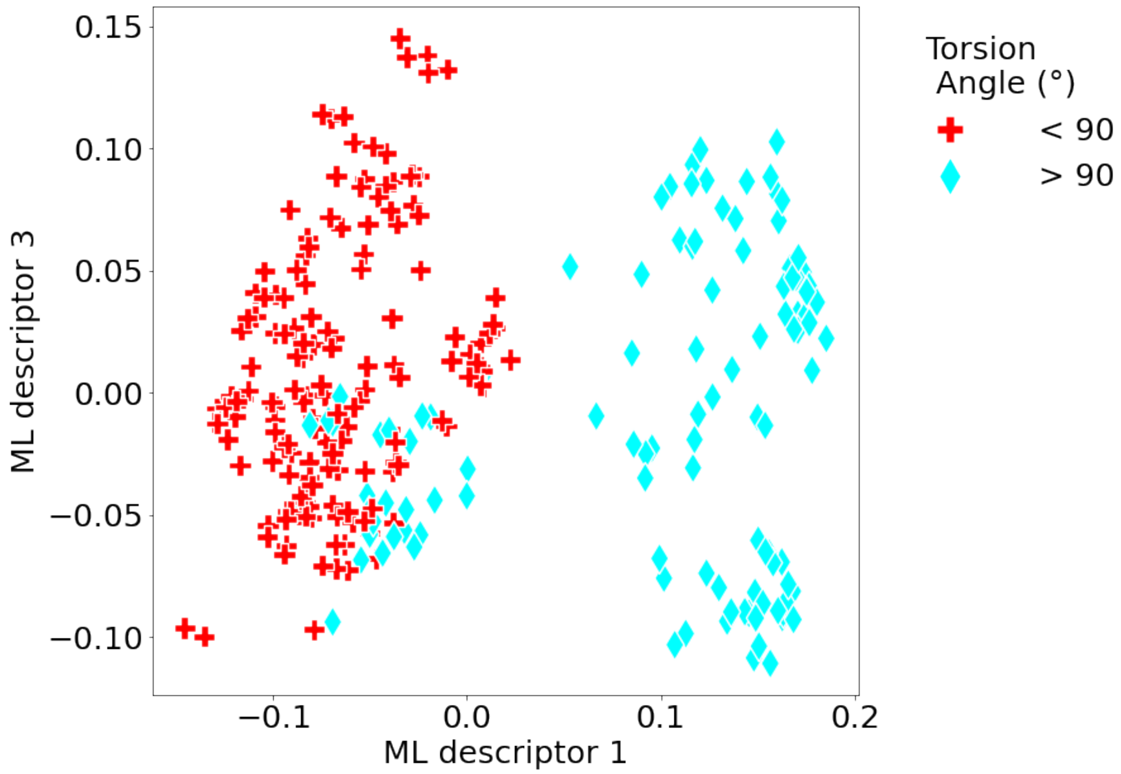

Fig. S9: ROY structure set plotted by the first and third ranked ML descriptors from the adapted kernel construction (4 Å SOAP cut-off) - coloured by whether on not a key intramolecular torsion in the underlying molecule is acute

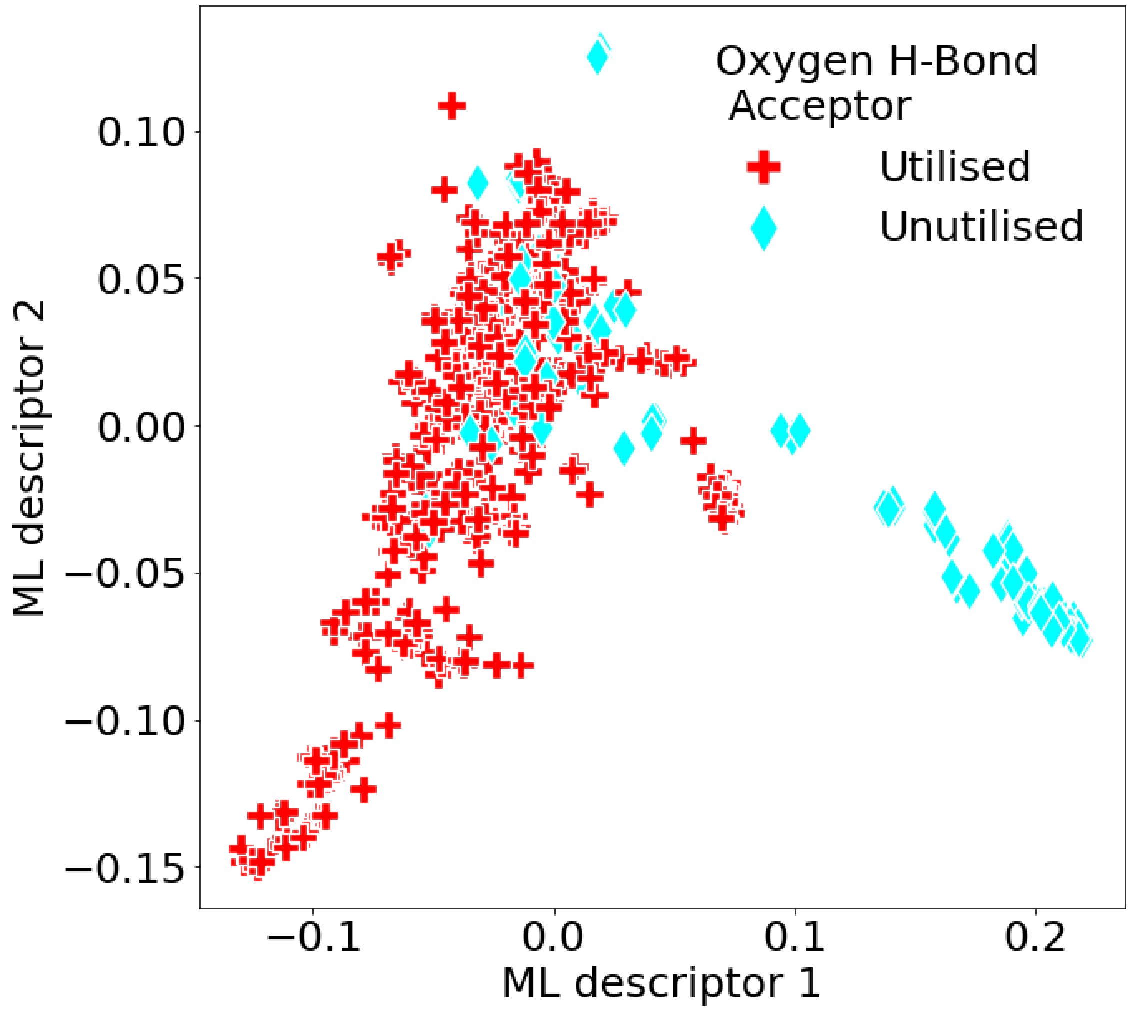

Fig. S10: Galunisertib structure set plotted by the top two ranked ML descriptors from the adapted kernel construction (4 Å SOAP cut-off) - coloured by whether on not the oxygen hydrogen bond acceptor is used

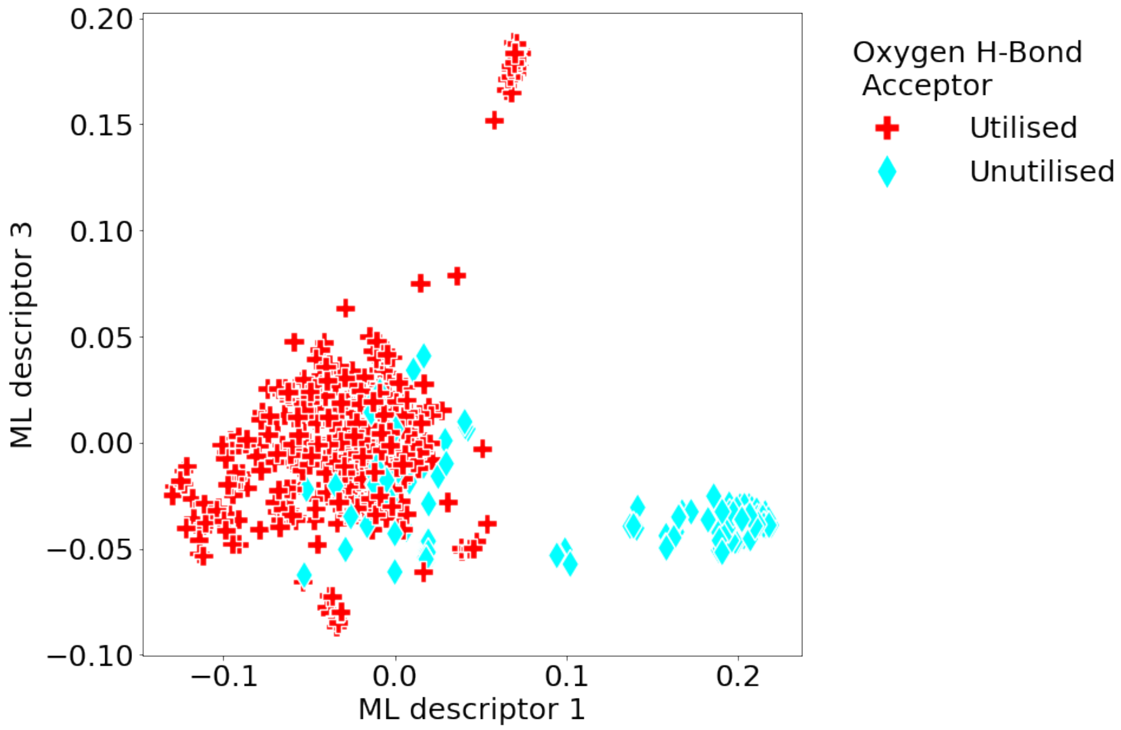

Fig. S11: Galunisertib structure set plotted by the first and third ranked ML descriptors from the adapted kernel construction (4 Å SOAP cut-off) - coloured by whether on not the oxygen hydrogen bond acceptor is used

12 Visual Comparisons of ML-Intuitive Descriptor Relationship Strength

Figures S12 and S13 show examples of relationships between ML descriptors and the intuitive descriptors - demonstrating the same relationships using descriptors derived via average and adapted kernels. These preliminary results indicated that the adapted kernel may lead to ML descriptors more closely related to intuitive descriptors. In all cases shown here, the 4 Å SOAP cut-off radii were used, and the ML descriptor employed is that, of the top 5 ranked ML descriptors from the respective kernel, that showed the clearest visual relationship to the intuitive descriptor.

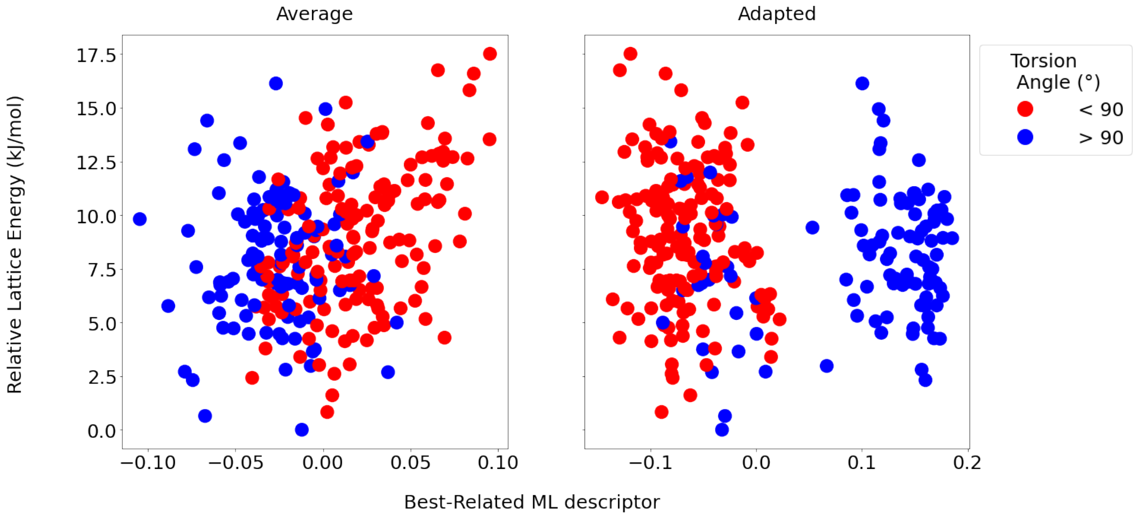

Fig. S12: 1D GCH landscapes (4 Å SOAP cut-off) of ROY from the average and adapted kernel constructions - coloured by the value of the molecular conformation binary classification descriptor. The ML descriptor plotted in each case is the kPCA component - of the top ranked five components in the eigenspectrum - that best related to the conformation classification

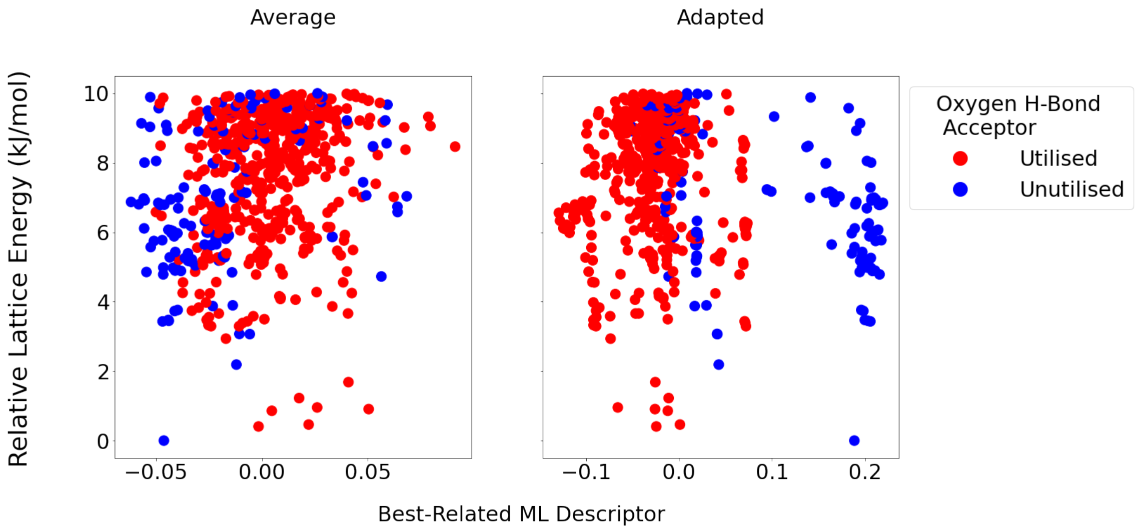

Fig. S13: 1D GCH landscapes (4 Å SOAP cut-off) of galunisertib from the average and adapted kernel constructions - coloured by whether on not the crystal structure uses the oxygen h-bond acceptor in intermolecular hydrogen bonding. The ML descriptor plotted in each case is the kPCA component - of the top ranked five components in the eigenspectrum - that best related to the hydrogen bonding classification

The poorer separation of classes when using the adapted kernel rather than the average appears to be maintained when considering secondary machine learned descriptors (Figures S14, S15, S16, S17)

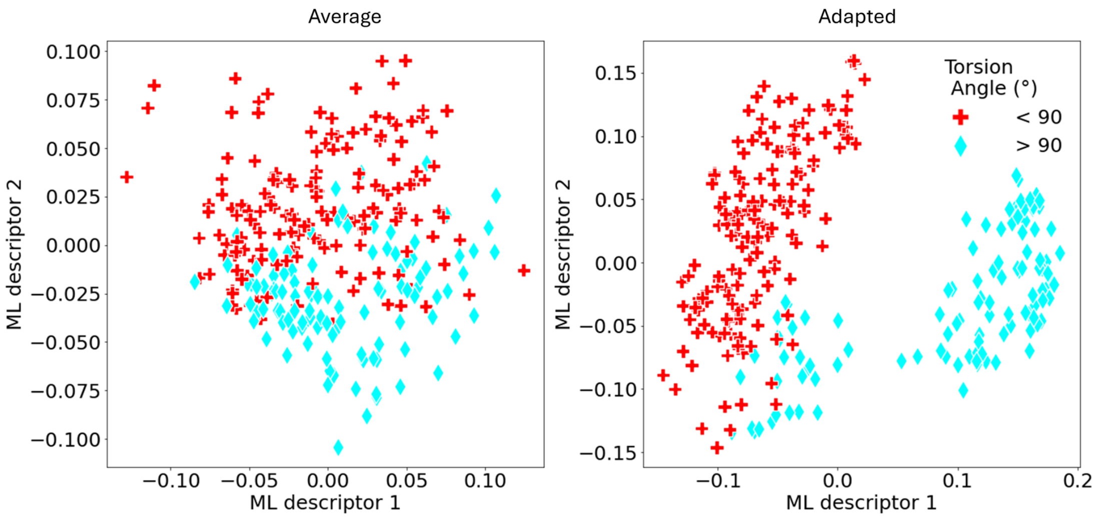

Fig. S14: Structure sets plotted by the top two ranked ML descriptors of ROY from the average and adapted kernel constructions (4 Å SOAP cut-off) - coloured by whether on not a key intramolecular torsion in the underlying molecule is acute

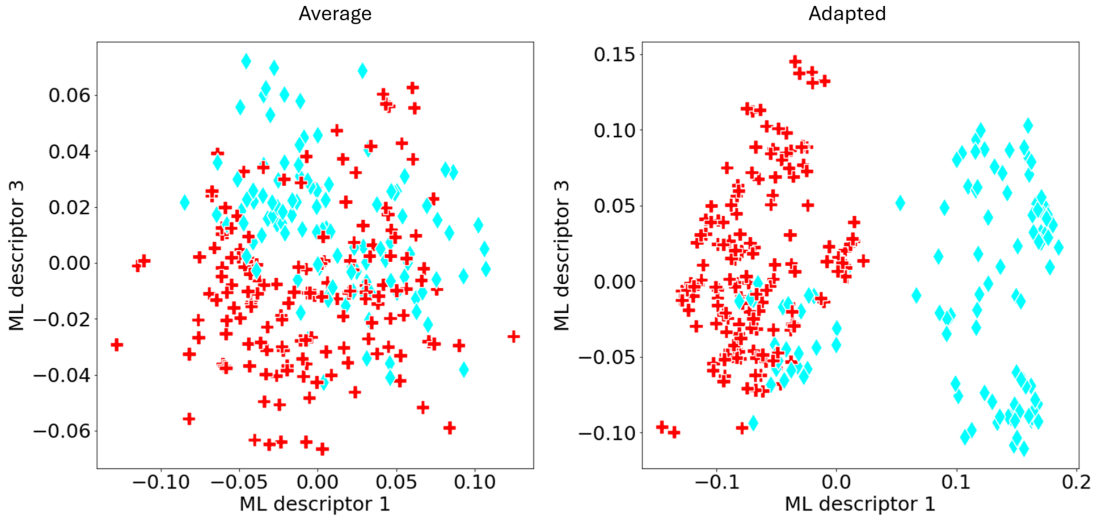

Fig. S15: Structure sets plotted by the first and third ranked ML descriptors of ROY from the average and adapted kernel constructions (4 Å SOAP cut-off) - coloured by whether on not a key intramolecular torsion in the underlying molecule is acute

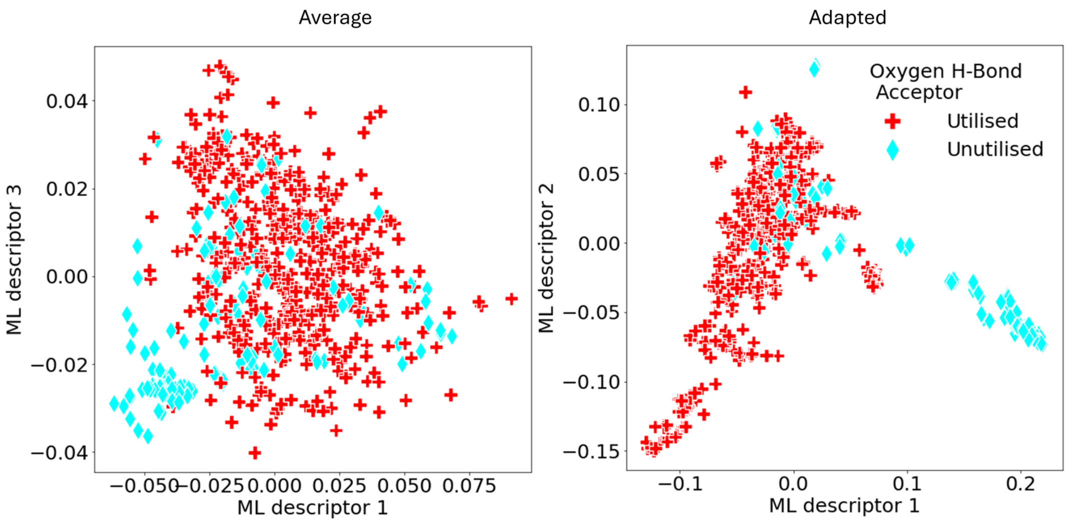

Fig. S16: Structure sets plotted by the top two ranked ML descriptors of galunisertib from the average and adapted kernel constructions (4 Å SOAP cut-off) - coloured by whether on not the oxygen hydrogen bond acceptor is used.

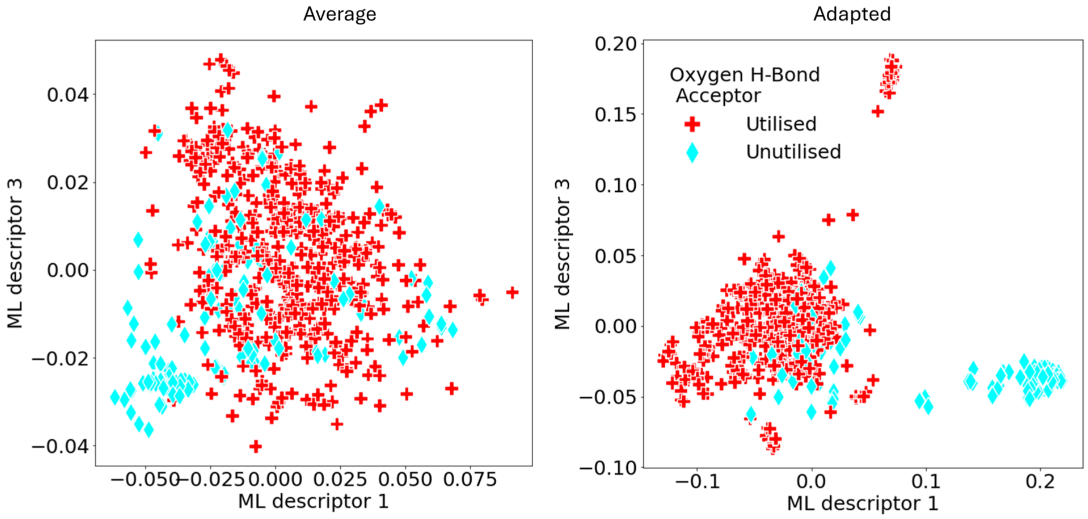

Fig. S17: Structure sets plotted by the first and third ranked ML descriptors of galunisertib from the average and adapted kernel constructions (4 Å SOAP cut-off) - coloured by whether on not the oxygen hydrogen bond acceptor is used.

13 Hydrogen Bonding Motif Search

The galunisertib molecule has four hydrogen bond acceptors and two equivalent hydrogen bond donors. Therefore, a hypothetical crystal structure can contain any combination (or none) of the four corresponding hydrogen bonding motifs.

To investigate the hydrogen bonding motifs in the structure set, a search was performed using *motif search* in Mercury - searching for hydrogen bonding between the donor and the four hydrogen bond acceptors. The 'molecular fragments' used in the motif search were designed to mimic those shown in discussion of galunesertib hydrogen bonding in reference [6]. The search was performed to identify all such intermolecular hydrogen bonds with lengths $\leq \sum(vDW radii) + 0.1$ Å. An illustration of the fragments defining this search can be seen in Figure S18.

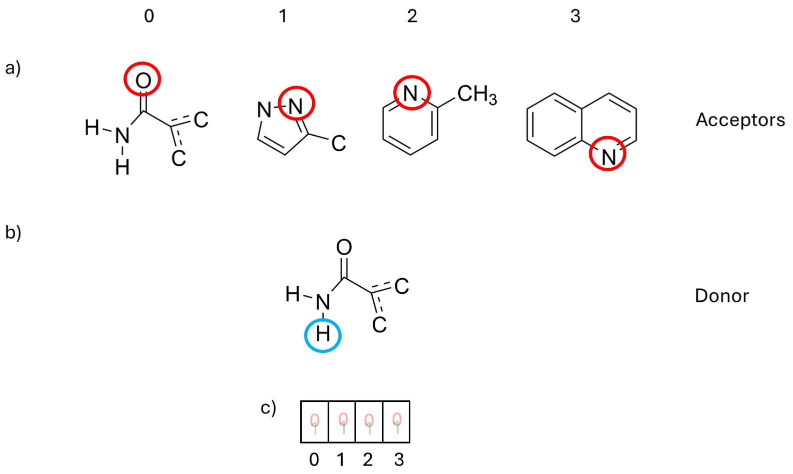

Fig. S18: Fragments of molecular structures used within the motif search to identify utilised hydrogen bond acceptors (a) and donors (b) within the crystal structures. The vector construction shown in c) indicates how class labels were applied to structures base upon the presence (1) or absence (0) of hydrogen bonds utilising the respective acceptors.

From the resulting data, structures were classified according to which motifs could be found within the crystal structure - using labels of concatenated binary values (0/1) to indicate the presence or absence in the crystal structure of hydrogen bonds with the corresponding motif (Figure S18(c)). Then, following initial investigations, this descriptor was reformulated as a binary classifier - describing whether (0) or not (1) each crystal structure contained intermolecular hydrogen bonding utilizing the oxygen hydrogen bond acceptor (Acceptor 0 in Figure S18 a.)

14 Removal of Unphysical Structures

Some structures in the chlorpropamide CSP set [14] were identified as having unphysically close intermolecular oxygen-hydrogen contacts. To trim such erroneous structures from the set. an energy cut-off was applied - designed to exclude anomalously high energy structures. From a set of 5000 single-point energy calculations, outliers were identified via the Inter Quartile Range (IQR) criterion:

$$x \in \text{outliers} \iff E_x > E_{cut}$$
$$E_{cut} = Q3 + 1.5 \times IQR \tag{2}$$

This resulted in an E_{cut} value of -17651.277 kJ/mol. As such all structures with total energy above this value were excluded and replaced in their respective training/test set by a randomly selected structure meeting the criterion for retention.

Based upon the original 5000 structures used to derive the retention criterion, the excluded structures correspond to 2.3% of the successful single-point energy calculations (Figure S19).

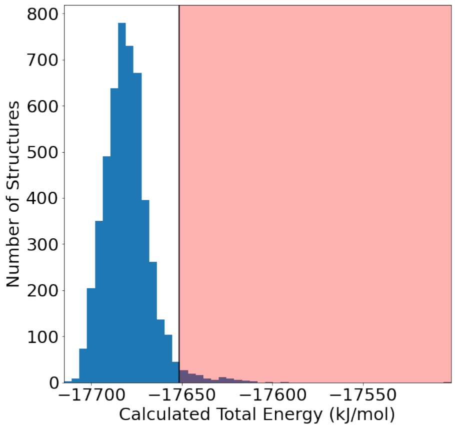

Fig. S19: Histogram displaying the distribution of calculated total energies for the first 5000 chlorpropamide crystal structures tested. The red shading indicates the region declared unrealistic - determined by use of an interquartile range criterion on the set of energies - and any structure whose energies is calculated to lie within this region is rejected.

Additionally, a minimal fraction of single-point energy calculations attempted failed due to convergence issues or other errors. The corresponding structures were rejected and replaced in their respective training or test sets by randomly selected structures.

15 Parity Plots for Energy Predictions

Figure S20 shows the parity plots (DFT energy vs ML predicted energy) of energy predictions for chlorpropamide at key points. N is the number of training structures used. a) shows the prediction behaviour of GPR using the average kernel with a 4 Å SOAP cut-off and b) shows the prediction behaviour of GPR using the adapted kernel with a 4 Å SOAP cut-off.

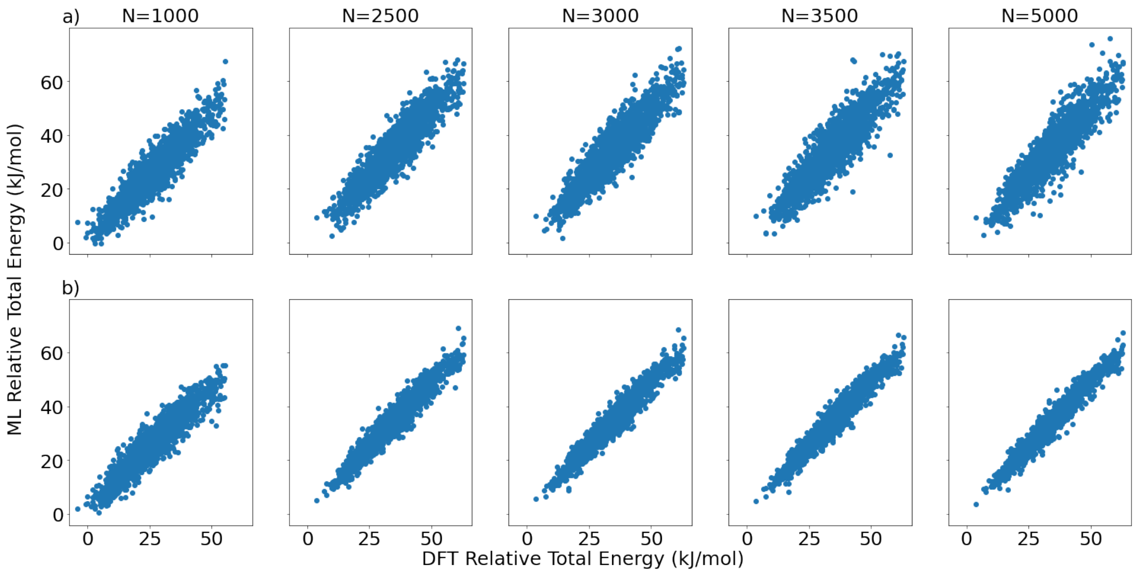

Fig. S20: Plots of DFT energy vs ML predicted energy for chlorpropamide at key points. N is the number of training structures used. Results shown for a) the average kernel and b) the adapted kernel. Both kernels used a 4 Å SOAP cut-off.

16 Graphs of Errors in Machine Learning of Energies with Smaller Training Sets

Figures S21 and S22 visualize the mean average errors and root mean square errors respectively of a GPR model working with a dataset of low energy subsets of the chlorpropamide structure set, alongside the single standard deviations of the cross validations.

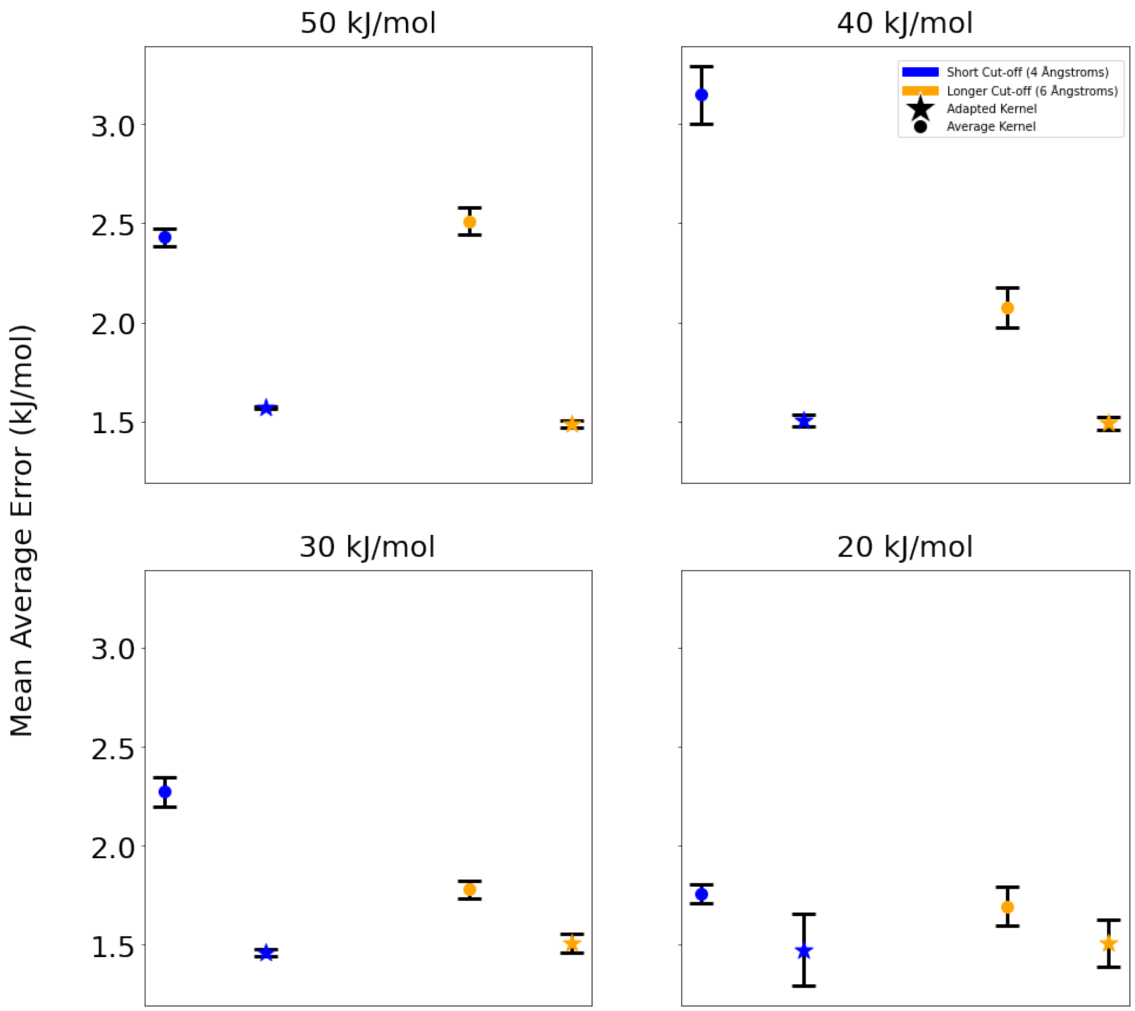

Fig. S21: Average MAE values of energy prediction on the extended chlorpropamide set, measured via cross-validation upon given low-energy subsets of the entire set of 8000 structures for which pDFT single point energies were calculated. Colour denotes the underlying descriptor cut-off radius and marker style indicates the kernel construction used in the GPR model. The error bars about each point display the uncertainty in the declared errors, with bars being of height (either side of the centre point) equal to one standard deviation of errors measured in cross validation.

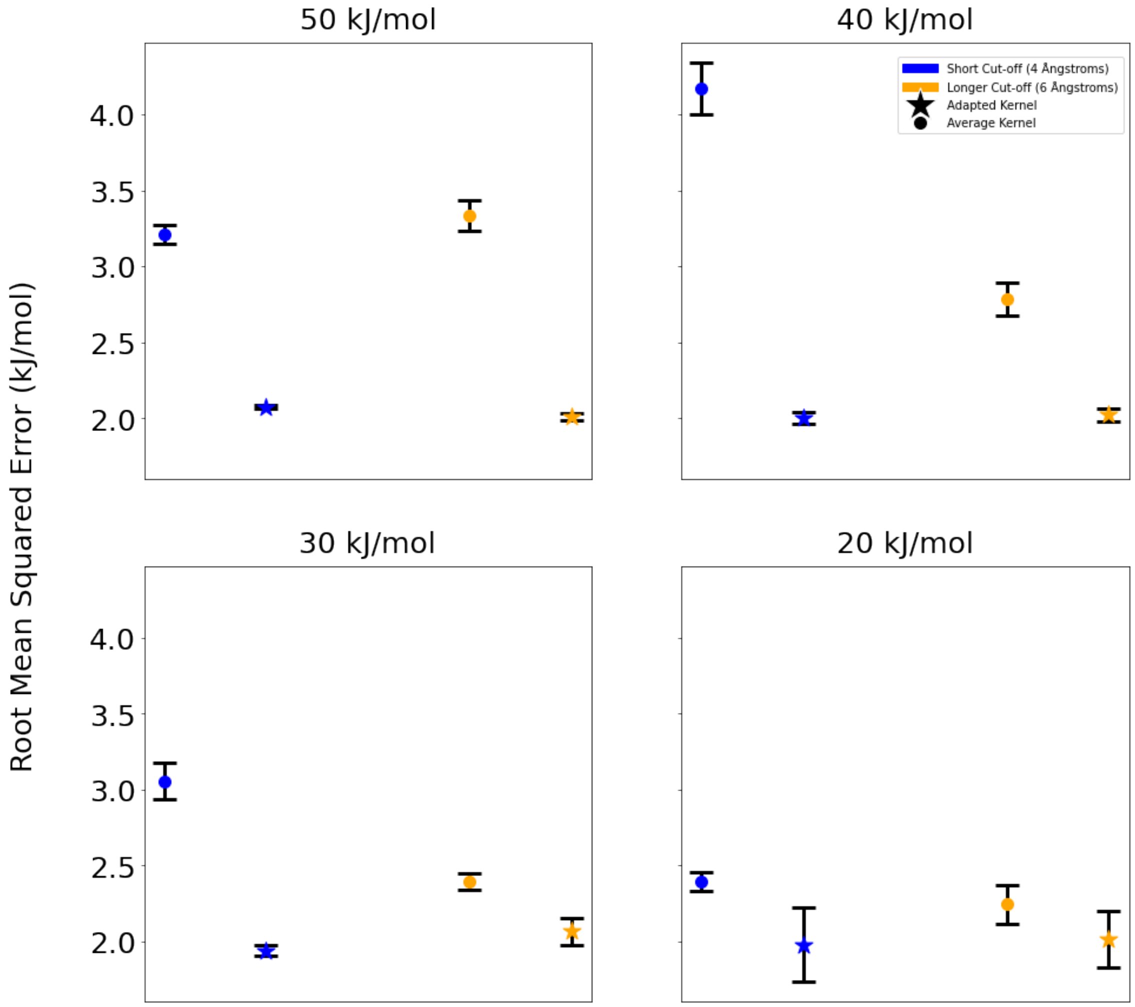

Fig. S22: Average RMSE values of energy prediction on the extended chlorpropamide set, measured via cross-validation upon given low-energy subsets of the entire set of 8000 structures for which pDFT single point energies were calculated. Colour denotes the underlying descriptor cut-off radius and marker style indicates the kernel construction used in the GPR model. The error bars about each point display the uncertainty in the declared errors, with bars being of height (either side of the centre point) equal to one standard deviation of errors measured in cross validation.

References

(1) Q. Zhu, J. Johal, D. E. Widdowson, Z. Pang, B. Li, C. M. Kane, V. Kurlin, G. M. Day, M. A. Little and A. I. Cooper, *J. Am. Chem. Soc.*, 2022, **144**, 9893–9901.

(2) G. J. O. Beran, I. J. Sugden, C. Greenwell, D. H. Bowskill, C. C. Pantelides and C. S. Adjiman, *Chem. Sci.*, 2022, **13**, 1288–1297.

(3) J. Weatherston, M. R. Probert and M. J. Hall, *Journal of the American Chemical Society*, 2025, **147**, PMID: 40132086, 11949–11954.

(4) R. A. Sykes, N. T. Johnson, C. J. Kingsbury, J. Harter, A. G. P. Maloney, I. J. Sugden, S. C. Ward, I. J. Bruno, S. A. Adcock, P. A. Wood, P. McCabe, A. A. Moldovan, F. Atkinson, I. Giangreco and J. C. Cole, *Journal of Applied Crystallography*, 2024, **57**, 1235–1250.

(5) J. Chisholm and S. Motherwell, *Journal*, 2005, **38**, 228–231.

(6) R. M. Bhardwaj, J. A. McMahon, J. Nyman, L. S. Price, S. Konar, I. D. H. Oswald, C. R. Pulham, S. L. Price and S. M. Reutzel-Edens, *J. Am. Chem. Society*, 2019, **141**, 13887–13897.

(7) D. H. Case, J. E. Campbell, P. J. Bygrave and G. M. Day, *J. Chem. Theory Comput.*, 2016, **12**, 910–924.

(8) mol-CSPy GitLab,`https://gitlab.com/mol-cspy/mol-cspy`,(Accessed: 2025-03-18).

(9) M. J. Frisch, G. W. Trucks, H. B. Schlegel, G. E. Scuseria, M. A. Robb, J. R. Cheeseman, G. Scalmani, V. Barone, B. Mennucci, G. A. Petersson, H. Nakatsuji, M. Caricato, X. Li, H. P. Hratchian, A. F. Izmaylov, J. Bloino, G. Zheng, J. L. Sonnenberg, M. Hada, M. Ehara, K. Toyota, R. Fukuda, J. Hasegawa, M. Ishida, T. Nakajima, Y. Honda, O. Kitao, H. Nakai, T. Vreven, J. A. Montgomery, J. E. Peralta, F. Ogliaro, M. Bearpark, J. J. Heyd, E. Brothers, K. N. Kudin, V. N. Staroverov, R. Kobayashi, J. Normand, K. Raghavachari, A. Rendell, J. C. Burant, S. S. Iyengar, J. Tomasi, M. Cossi, N. Rega, J. M. Millam, M. Klene, J. E. Knox, J. B. Cross, V. Bakken, C. Adamo, J. Jaramillo, R. Gomperts, R. E. Stratmann, O. Yazyev, A. J. Austin, R. Cammi, C. Pomelli, J. W. Ochterski, R. L. Martin, K. Morokuma, V. G. Zakrzewski, G. A. Voth, P. Salvador, J. J. Dannenberg, S. Dapprich, A. D. Daniels, Ö Farkas, J. B. Foresman, J. V. Ortiz, J. Cioslowski and D. J. Fox, *Gaussian 09 Revision A.2*, 2009.

(10) S.R.Cox, L-Y.Hsu, D.E.Williams, *Acta Crystallogr.* 1981,**37**,293-301.

(11) D. S. Coombes, S. L. Price, D. J. Willock and M. Leslie, *The Journal of Physical Chemistry*, 1996, **100**, 7352–7360.

(12) C. R. Groom, I. J. Bruno, M. P. Lightfoot and S. C. Ward, *Acta Crystallogr., Sect. B*, 2016, **72**, 171–179.

(13) T. Stolar, J. Alić, I. Lončarić, M. Etter, D. Jung, O. K. Farha, I. Đilović, E. Meštrović and K. Užarević, *CrystEngComm*, 2022, **24**, 6505–6511.

(14) M. R. Ward, C. R. Taylor, M. T. Mulvee, G. I. Lampronti, A. M. Belenguer, J. W. Steed, G. M. Day and I. D. H. Oswald, *Crystal Growth & Design*, 2023, **23**, 7217–7230.

(15) J. Moellmann and S. Grimme, *The Journal of Physical Chemistry C*, 2014, **118**, 7615–7621.

(16) A. M. Reilly and A. Tkatchenko, *The Journal of Chemical Physics*, 2013, **139**, 024705.
